# Molecular Mechanisms and Determinants of Innovative Correction Approaches in Coagulation Factor Deficiencies

**DOI:** 10.3390/ijms20123036

**Published:** 2019-06-21

**Authors:** Dario Balestra, Alessio Branchini

**Affiliations:** Department of Life Sciences and Biotechnology, University of Ferrara, 44121 Ferrara, Italy

**Keywords:** coagulation factor deficiencies, gene therapy, TALEs, CRISPR activation, RNA-based correction approaches, modified U1 snRNA, ribosome readthrough, chaperone-like compounds

## Abstract

Molecular strategies tailored to promote/correct the expression and/or processing of defective coagulation factors would represent innovative therapeutic approaches beyond standard substitutive therapy. Here, we focus on the molecular mechanisms and determinants underlying innovative approaches acting at DNA, mRNA and protein levels in inherited coagulation factor deficiencies, and in particular on: (i) gene editing approaches, which have permitted intervention at the DNA level through the specific recognition, cleavage, repair/correction or activation of target sequences, even in mutated gene contexts; (ii) the rescue of altered pre-mRNA processing through the engineering of key spliceosome components able to promote correct exon recognition and, in turn, the synthesis and secretion of functional factors, as well as the effects on the splicing of missense changes affecting exonic splicing elements; this section includes antisense oligonucleotide- or siRNA-mediated approaches to down-regulate target genes; (iii) the rescue of protein synthesis/function through the induction of ribosome readthrough targeting nonsense variants or the correction of folding defects caused by amino acid substitutions. Overall, these approaches have shown the ability to rescue the expression and/or function of potentially therapeutic levels of coagulation factors in different disease models, thus supporting further studies in the future aimed at evaluating the clinical translatability of these new strategies.

## 1. Blood Coagulation and Hemorrhagic Disorders

Blood coagulation is a finely tuned system involving a series of strictly controlled cellular and molecular events that ultimately lead to the formation of a stable clot. In the first response to a vessel damage, the von Willebrand factor (vWF)-mediated adhesion and aggregation of platelets, which also interact with subendothelial collagen, provide the key negatively charged surfaces acting as scaffolds for coagulation reactions. Plasma coagulation factors represent the soluble molecular components involved in the so-called coagulation cascade, in which the central event is the conversion of prothrombin to thrombin, the central player participating in clot formation as well as in a series of feedback reactions that potentiate the first pro-coagulant event. Overall, the dynamic and mutual interaction of these molecular and cellular components, which has been well described through a cell-based model, ensures proper hemostasis and prevents major blood loss [1].

**Figure 1:**
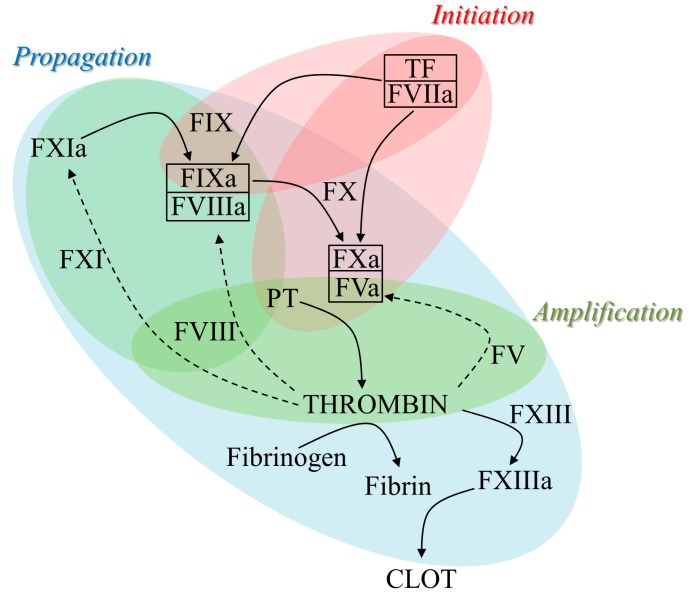
The series of enzymatic reactions in the coagulation cascade. Schematic representation of the coagulation cascade, showing the several direct (black rows) or feedback (dotted rows) reactions that can be subdivided into initiation (red), amplification (green) and propagation (blue) phases, ultimately leading to the fibrin clot. Boxed items indicate the interaction of active enzymes (FVIIa, FXa, FIXa) with their cofactors (TF, FVa, FVIIIa). F, factor; a, activated form; PT, prothrombin; TF, tissue factor.

The series of reactions leading to the sequential activation of coagulation factors, through the conversion of circulating zymogens to active serine proteases, involves several pro-coagulant enzymes and cofactors as well as proteins with structural roles. Briefly, after a vessel wall injury, the initiation phase of coagulation is triggered through the interaction of the exposed tissue factor (TF) with activated factor (F) VII (FVIIa) that converts FIX to FIXa and FX to FXa. The latter is the key protease that, in complex with cofactor FV in its activated form (FVa), is directly involved in the generation of thrombin. In these sequential steps, thrombin also boosts its own production through the activation of platelets and feedback reactions known as the amplification and propagation phases, during which a crucial feedback loop activates FXI (FXIa) that in turn increases the amount of FIXa. The interaction of FIXa with the activated form of the key cofactor FVIII (FVIIIa) strongly boosts the production of FXa that, in association with FVa, further drives the so-called thrombin burst responsible for the large-scale production of thrombin necessary to produce the final clot. Indeed, the great amount of generated thrombin converts fibrinogen to insoluble fibrin as well as activates the transglutaminase FXIII (FXIIIa), which stabilizes the clot by cross-linking the fibrin chains (Figure 1).

In this scenario, a functional defect of one of the pro-coagulant players alters the production of thrombin, which in turn affects the formation or stability of the clot, thus resulting in bleeding disorders. Coagulation deficiencies, characterized by a different degree of severity depending on the affected protein and the corresponding residual levels, show either X-linked (FIX/FVIII deficiency) or autosomal (fibrinogen, prothrombin, FV, FVII, FX, FXI, FXIII deficiency) inheritance patterns. The genetic and clinical features, including treatment options, of the above-mentioned coagulation deficiencies are summarized in Table 1.

The X-linked coagulation deficiencies, namely those of FVIII (hemophilia A) and FIX (hemophilia B), are characterized by a heterogeneous pattern of mutations as the cause of the corresponding defects. In particular, together with missense mutations being the most frequent gene alterations, in the mutational pattern of both deficiencies, a relatively frequent cause is ascribable to nonsense and splice site mutations, deletions/insertions and promoter mutations. In addition, the inversion of intron 1 or 22 in the *F8* gene is responsible for approximately a half of severely affected hemophilia A patients [2,3,4,5,6]. Since the activity of upstream factors is in the normal range, hemophilia is a defect in clot stabilization, rather than in coagulation itself, due to the inefficient FIX/FVIII-dependent feedback loop responsible for the large-scale production of thrombin.

The autosomal recessive deficiencies of fibrinogen, prothrombin, FV, FVII, FX, FXI and FXIII, also referred to as rare bleeding disorders, display a variable prevalence ranging from 1:500,000 (FVII deficiency) to 1:2,000,000 (prothrombin and FXIII deficiency). The clinical features of these disorders range from asymptomatic, as observed for heterozygotes with approximately half-normal levels of coagulation factors, to severe phenotypes typical of homozygotes or compound heterozygotes. Causative gene defects can be classified into mutations affecting protein biosynthesis/secretion, thus resulting in low/very low antigen or activity in plasma, or leading to reduced or near-normal secretion of a dysfunctional or devoid-of-function protein. Generally, in the mutational pattern of these deficiencies, missense mutations are the most frequent (50–80%), splicing and nonsense mutations account for 5–15% and insertions/deletions represent <15% of the total, with the exception of fibrinogen, FV and FXIII deficiencies (20–30%) [7,8]. Importantly, the complete absence of FVII, FX and prothrombin is virtually incompatible with life due to their pivotal role in triggering key steps of coagulation, namely the initiation phase (FVII) or the generation of thrombin as activator (FX) or zymogen (prothrombin) [9,10,11,12]. In the deficiencies of fibrinogen, FV and FXIII, symptoms associated with low or very low levels are heterogeneous and range from severe to life-threatening. Finally, at variance from the other coagulation disorders, the bleeding phenotype of FXI deficiency, the prevalence of which is higher in Ashkenazi Jews and French Basques, correlates with the site of injury, with the risk of bleeding being high in sites with high activity of the fibrinolytic pathway responsible for clot lysis upon healing [13].

The treatment mainstay for coagulation factor disorders is based on replacement therapy, namely the administration of the defective factor for prophylaxis or “on-demand” interventions, with either plasma-derived or recombinant disease-specific protein concentrates [14]. In addition, other non-specific products such as fresh frozen plasma (FFP), a pool of plasma obtained from blood of healthy donors, or prothrombin complex concentrate (PCC), plasma fractions enriched with prothrombin, FIX and FX with or without FVII, are still possible options. The potential side-effects of FFP, such as hypervolemia due to the large volume required to achieve efficacy from the low starting factor concentration [15], or of PCC, such as thrombotic complications [16], limit their use for bleeding disorders with no alternative. Otherwise, specific factor concentrates are the treatment of choice. Factor-specific treatments cover mainly hemophilia A and B, but also other disorders such as fibrinogen deficiency (plasma-derived [pd]fibrinogen), FVII deficiency (pdFVII concentrate), FX deficiency (combined FIX/FX concentrate or a recent high-purity pdFX [17]), and FXI deficiency (pdFXI product) have disease-specific treatments. Regarding FXIII deficiency, together with a pFXIII concentrate, a new recombinant product (rFXIII-A2) is now available [18,19]. For prothrombin and FV deficiencies, a specific treatment is not yet available, even if a FV concentrate has recently been developed and has shown the ability to correct coagulation parameters in laboratory assays [20]. Particular attention deserves recombinant (r)FVIIa, the advent of whivh has represented an enormous advancement, together with being the optimal therapy for FVII deficiency, as by-passing agent in hemophilia A and B patients with inhibitors. Indeed, the development of inhibitory antibodies to the infused factor represents the most severe complication in replacement therapy, particularly in hemophilias (A, ~30% of patients; B, 3–5%) [21,22], in which the use of rFVIIa is pivotal to bypass the deficient FIX/FVIII-dependent pathway and increase thrombin generation by restoring or boosting the FX-to-FXa conversion [23,24]. On the other hand, one of the main drawbacks of replacement therapy is the short half-life of coagulation factors once infused, which limits the efficacy of treatment. Novel agents such as long-acting molecules with improved half-life have been developed by means of different strategies including the fusion technology [25,26], and pro-coagulant factors such as FVIIa, FX or FV with enhanced biological properties have been engineered for the treatment of hemophilia [27]. Finally, for hemophilia patients, gene therapy is a potential new option with growing evidence of efficacy (excellently reviewed in [27]), even if very interesting data have been obtained in two animal models of FVII deficiency [28,29].

## 2. Overview of Correction Approaches at DNA, mRNA and Protein Levels

The quality of life, as well as life expectancy, of patients with inherited coagulation factor disorders has been tremendously ameliorated by the development of replacement therapy in response to bleeding episodes or for prophylaxis [30]. These aspects also took advantage of protein modifications and/or engineering through fusion strategies, which extend the overall half-life of the infused therapeutic protein, the main drawback of substitutive therapy [25,31]. Fusion strategies such as those exploiting albumin, in which half-life of the target albumin-fused coagulation factor is prolonged by the albumin recycling pathway mediated by the neonatal Fc receptor, will not be the focus of this review. However, three key coagulation factors, namely, factor FVII [32], FIX [33] and FX [34], with different requirements in terms of fusion strategy, have been produced and tested in different models and trials, with FIX having approached the market.

However, new therapeutic approaches, and particularly gene therapy, have been developed to achieve a significantly prolonged, or permanent, production of the missing protein at therapeutic levels. Knowledge of the molecular genetics of inherited coagulation disorders, in particular X-linked hemophilia A and B [2], but also the rare autosomal FVII [35] and FX [36] deficiencies, favored their investigation. Moreover, coagulation factor deficiencies are ideal models to evaluate the efficacy of gene therapy and other innovative therapeutic approaches, because even tiny increases in coagulation factor levels would ameliorate the clinical phenotype [37]. Various outstanding reviews have reported the major advances of substitutive gene therapy at pre-clinical and clinical levels [27,38,39,40], including the delivery of the wild-type protein into patients’ cells, with a stable endogenous expression of the missing coagulation factor. Hemophilia B has been the archetypal coagulation disorder for gene therapy attempts due to the small size of the cDNA encoding the FIX protein. The coding cassette, the expression of which is mediated by a small but efficient liver-specific promoter, has been delivered by adeno-associated viral (AAV) vectors. In particular, the delivery of FIX by a recombinant AAV serotype 8 vector, ensuring high liver tropism, in severe hemophilia B patients resulted in the stable and multi-year expression of human FIX, and in a significant reduction in bleeding episodes as well as of the use of the therapeutic FIX protein [41]. The transient increase in liver enzymes, associated with AAV8 infection of liver cells triggering immunological response, has been overcome with a short course of prednisolone. Not surprisingly, advances in hemophilia B gene therapy increased research in other coagulation disorders, even in the rare ones transmitted as autosomal recessive traits. This is the case of FVII deficiency, in which AAV-mediated gene therapy has been evaluated in a natural canine model through the expression of canine FVII transgene as well as exploiting codon-optimized human FVII in adult macaques, with very promising data on the longevity and stability of therapeutic levels of FVII expression in the absence of side-effect complications [28,29,42,43].

Here, this review is focused on the molecular mechanisms and determinants of alternative approaches and aimed at achieving coagulation factor expression. In the first part, great attention will be given to interventions at the DNA level, including gene editing. These approaches seem to represent a sort of “holy grail” for molecular biologists and geneticists, since they promise to correct all inherited diseases without the common bottleneck of classical gene therapy represented by the size of the replaced gene. Generally, these gene therapy approaches rely on engineered nucleases that are able to recognize and cut specific DNA sequences. After DNA damage, the innate cellular DNA repair pathways occur, and thus the disease-causing variant is removed from cells. Another category of molecules is represented by trans-activating factors, in which an engineered DNA-binding domain is fused to a transcriptional activator domain, thus generating a specific transcription factor able to counteract the silencing effects of promoter mutations.

In the second part, we will focus on the modulation of pre-messenger RNA (pre-mRNA) processing, also known as splicing. To this aim, variants of small nuclear RNAs (snRNAs) as well as antisense oligonucleotides (AONs) are commonly used to modulate splicing and represent complementary strategies to target sequences on pre-mRNA, directly masking splicing regulatory elements or recruiting additional splicing factors. The exploitation of AONs for therapeutic purposes has been reported in other excellent reviews [44,45,46] and variants of snRNAs, particularly U1 and U7 snRNAs, have been reported in human disease models other than coagulation factor deficiencies [47,48]. Differently, here we report the exploitation of U1snRNA variants to rescue splicing abnormalities caused by splicing changes at the donor and acceptor splice sites.

In the third part, we shall focus on interventions at the translational and post-translational levels. In particular, we provide details on the molecular determinants of ribosome readthrough targeting nonsense variants, another mRNA-based approach for the potential treatment of inherited coagulation factor disorders, and on the use of small compounds with chaperone-like activity to correct protein folding impaired by amino acid substitutions.

It is worth noting that the second and third sections of this review are aimed at targeting pre-mRNA and mature mRNA. Indeed, the targeting of RNA instead of DNA provides potential advantages over substitutive gene therapy. First, it allows the restoration of gene expression without altering its regulation and occurs only in the physiological cells and tissues. Moreover, the mRNA-directed approaches have the potential to circumvent a key limitation due to the large size of disease genes, thus preventing viral delivery, and could also be effective to address dominant negative disease mechanisms [49].

**Figure 2:**
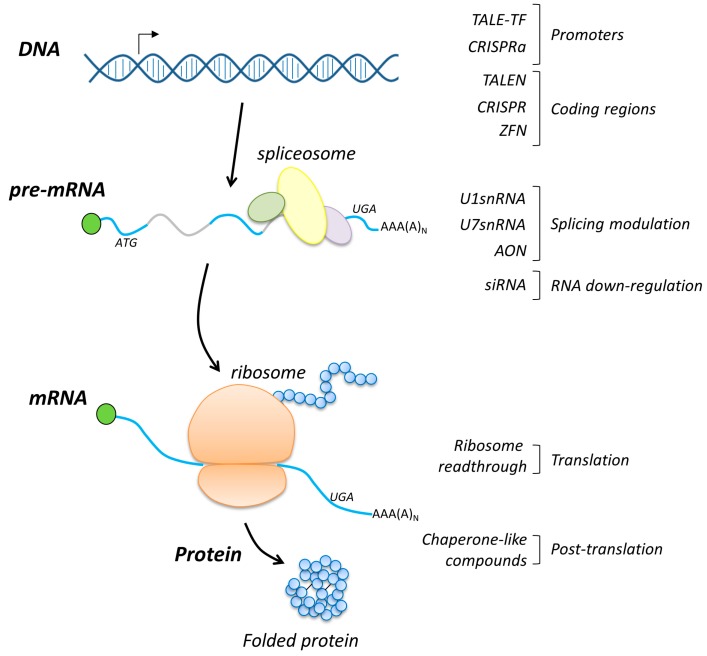
Overview of the interventions at the DNA, RNA and protein levels. Schematic representation of the DNA–RNA–protein flow (**left**) as well as of the corresponding molecular approaches with the indicated level at which each strategy works (**right**). In the pre-mRNA scheme, exons and introns are indicated in cyan and grey, respectively.

Overall, the application of these alternative approaches in several cellular models and in selected mouse models of disease has revealed their therapeutic potential, thus supporting further studies aimed at demonstrating the clinical translatability of specific molecules for individualized therapies. A schematic representation summarizing the above-mentioned approaches is depicted in Figure 2.

## 3. Rescue of Coagulation Factor Genes at the DNA Level

Gene editing approaches are based on engineered nucleases able to recognize and cut specific DNA sequences into the cellular genome. The DNA damage triggers the innate cellular DNA repair pathways, including the non-homologous end joining (NHEJ) and homology directed repair (HDR), to introduce a targeted modification in the genome and to achieve the genetic correction of mutations in chromosomes. To date, four nuclease families have been engineered and applied to create new gene correction strategies: meganucleases (MGNs), zinc-finger nucleases (ZFNs), transcription activator-like effector nucleases (TALENs) and clustered regulatory interspaced short palindromic repeats associated with RNA-guided Cas9 (CRISPR-Cas9) nucleases [50]. Albeit with different efficiencies, these nucleases can be modified to precisely introduce a double-strand break (DSB) in the target locus, which is normally repaired by either NHEJ or HDR. Between the two repair mechanisms, the NHEJ is the prevalent in quiescent cells and involves the introduction of small insertions/deletions due to direct ligation of DNA ends. For therapeutic purposes, the NHEJ mechanism can be exploited to excise deleterious mutations, or restore the reading frame of the coding gene [51]. On the other hand, HDR occurs mostly in dividing cells and, through a donor DNA with homologous sequences to those adjacent to the DSB, can replace the target mutation. For therapeutic purposes, DSB can be exploited to achieve a repair of a nucleotide change or to knock-in an entire cDNA [52]. The insertion could be at the endogenous locus or within a genomic region where the transgene can be integrated without altering other gene functions.

It is worth noting that gene editing approaches are able to induce the permanent modification of the target genome, thus being the elective strategies for the creation of cell and animal models. With these tools it is possible to design a specific gene deletion (knock-out), insertion (knock-in), excision and virtual correction of all genetic variants.

### 3.1. Gene Replacement

For hemophilia B, various efforts have been carried out to demonstrate the feasibility of genome editing for therapeutic purposes. In particular, two different strategies have been applied, with ZFNs and CRISPR/Cas9 approaches exploited in mice [53,54,55], and in induced pluripotent stem cells (iPSCs) from patients.

**Figure 3:**
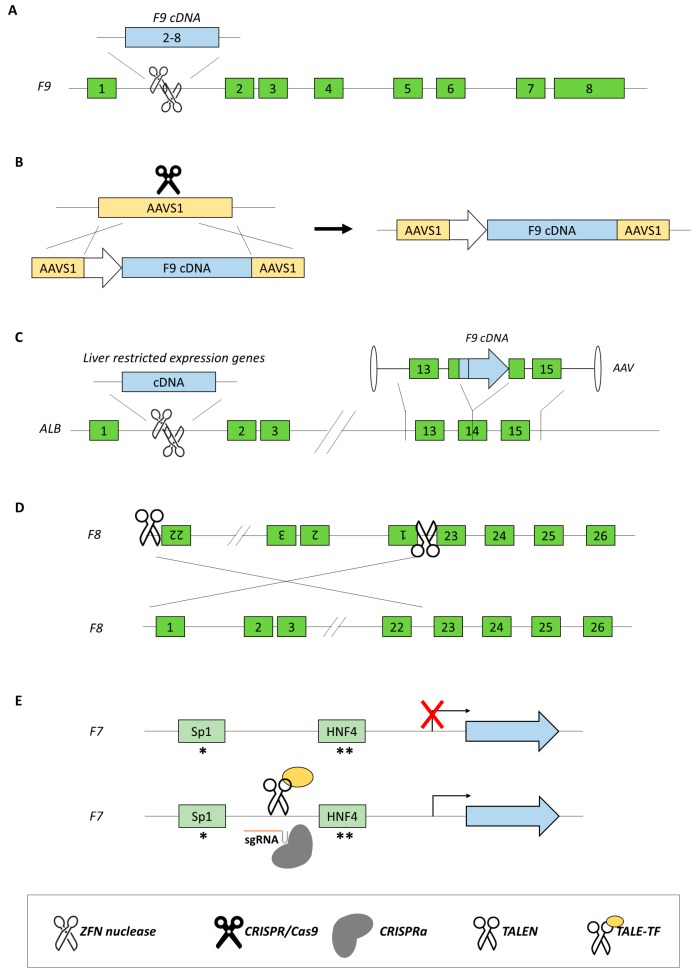
Gene editing and transcription modulation-based approaches for coagulation factor deficiencies. Approaches aimed at correcting and/or modulating the expression of coagulation factor genes through ZFNs (**A**), cleavage by CRISPR/Cas9 (**B**), engineering of the albumin locus to drive *F9* gene expression (**C**), correction of intron 22 inversion in *F8* gene (**D**), and TALE-TF or the CRISPRa systems leading to an increase in luciferase (reporter constructs) or endogenous activity due to *F7* promoter transactivation (**E**). Asterisks represent the c.-94C>G (*) and c.-61T>G (**) nucleotide changes.

In mice, the delivery of AAV8-ZFNs and a corrective cDNA (promoterless *F9* exons 2–8 bearing a splice acceptor and a poly A signal flanked by homology arms, Figure 3A) was able to induce gene repair through HDR, and the correction resulted in the rescue of clotting times. Moreover, partial hepatectomy showed stable genome modification, in contrast to episomal AAV-mediated *F9* transgene delivery that decreased to almost background FIX levels after surgery. Analyses in adult mice showed the sustained expression of human FIX, averaging 23% of normal levels at week 60 [54].

Since the coding cassette of CRISPR/Cas9, the guide (g)RNA and the donor DNA do not fit into a single AAV particle, initial attempts based on CRISPR/Cas9 to correct hemophilia B exploited the hydrodynamic injection of linearized DNA [55]. Notably, the delivery of DNA led to the genome editing of the aberrant allele and the FIX concentration rose to an average of 3.39 times the concentration in control mice, with a remission of coagulation deficiency.

Other studies [56,57] demonstrated the therapeutic potential of the gene replacement strategy by exploiting the CRISPR/Cas9 technology in mice, in which the introduction of a human FIX (hFIX) coding cassette into ex vivo cells followed by their delivery into an immune-deficient mice resulted in the secretion of hFIX into mouse blood.

### 3.2. Gene Addition

The need for high expression levels for therapeutic purposes but also for liver-restricted expression to reduce off-target effects and inhibitor risk requires the development of regulatory sequences (promoter and enhancer elements) that sometimes exceed the size of packaging vectors like AAV. Therefore, one strategy to overcome this limitation is offered by the possibility to insert the therapeutic transgene, without any promoter sequence, downstream of a highly expressed and liver-specific protein such as albumin. It is worth noting that a tiny reduction in albumin expression does not appear to be detrimental. In this light, the insertion by AAV8-ZFN into the highly expressed albumin locus of promoterless *F8* and *F9* transgenes in hemophilic mice resulted in the therapeutic and long-term expression of both human FVIII and FIX [58] (Figure 3B). Another study [59], delivering FIX into the albumin gene in new born and adult mice (Figure 3C), but exploiting normal HDR without inducing DSB, resulted in gene correction in 0.5% of tested hepatocytes, with FIX expression reaching levels of up to 20%. Overall, these studies clearly indicate that some genomic loci, like the albumin locus, are both permissive and amenable for transgene integration. Moreover, they indicated that exploiting the physiological HDR process (nucleases-free approach) can have therapeutic implications, without the problematic off-target effects due to the presence of plasmid-coded nucleases.

In another study [60], coagulation FIX-deficient mice were created by the disruption of the *F9* coding gene through the AAV8-mediated delivery of the Cas9 nuclease and proper gRNA(s) into the liver. In this genomic context, different approaches have been exploited to correct the induced phenotype. In particular, the authors showed that the insertion of the target sequence at the DSB using NHEJ was more effective in increasing plasma FIX levels compared with HDR, even in mouse neonates. Interestingly, the portion of *F9* coding sequence spanning exon 2 through 8 was inserted into intron 1, allowing the potential correction of all FIX mutations occurring in *F9* gene.

### 3.3. New Approaches for Inversions

The genome editing approaches to restore proper FVIII expression have been more challenging mainly because of the size of *F8* cDNA and its inherently inefficient protein biosynthesis. Moreover, complex mutations as large inversions and duplications worsened this scenario. Among all mutations identified in hemophilia A patients, the inversions of intron 1 and of intron 22 are the most frequent genomic rearrangements accounting for 50% of patients [6]. Initially, an approach based on TALEN nucleases was used to invert a 140-kbp chromosomal segment spanning the portion of the *F8* gene in iPSCs to create a cell line mimicking hemophilia A. Then, the same TALEN pair was used to revert the inverted segment back to its normal orientation, providing the first proof-of-concept [61]. Later on, a different study, by exploiting a Cas9-based approach with target sites on either side of the inversion (Figure 3D), demonstrated the possibility to revert a ~600 kbp inversion in iPSCs derived from hemophilia A patients. The corrected iPSCs showed the ability to express a functional FVIII protein after differentiation into epithelial cells, and their injection corrected the hemophilic phenotype in mice [62].

### 3.4. New Approaches for Point Mutations

Among all the mutations identified in human inherited diseases, point mutations are the most represented. Therefore, the ability to efficiently correct point mutations has huge therapeutic implications. In the hemophilia B field, a recent study [63] exploiting a Cas9-based approach demonstrated the possibility to correct disease-causing point mutations in endogenous *F9*. Through the delivery of Cas9 nuclease and donor DNA as naked DNA molecules, to liver tissue by hydrodynamic injection, single-stranded DNA oligonucleotides (ssODNs) and plasmid donor-mediated HDR efficiency of 0.56% and 1.55% were respectively attained. Surprisingly, the lower HDR efficiency restored hemostasis in hemophilic mice. This study is considered as an in vivo genome-editing strategy with a potential therapeutic implication, although this approach is likely not applicable to human subjects.

### 3.5. Approaches for Promoter Transactivation

An alternative approach to rescue gene expression by acting at the DNA level is transcriptional activation by engineered transcription factors. In particular, TALE DNA-binding domains linked to a transcriptional activator domain (VP64) to create a TALE-TF have been exploited to efficiently correct promoter mutations, as demonstrated for coagulation FVII (Figure 3E) [64]. In this study, severe coagulation FVII deficiency was mimicked in reporter constructs by introducing c.-94C>G or c.-61T>G mutations, whose detrimental effect on *F7* promoter activity is caused by impaired binding of Sp1 or HNF-4 transcription factors [65,66]. In hepatoma HepG2 cells, expression studies with the luciferase reporter gene under the control of *F7* promoter identified a TALE-TF module able to specifically rescue the reporter gene expression in the presence of both promoter variants (>100-fold). Interestingly, the same module also strengthened (20- to 40-fold) the expression of the wild-type *F7* promoter. When translated into hepatic HepG2 cells and AAV-transduced primary hepatocytes, the TALE-TF module increased endogenous *F7* mRNA and protein expression (2- to 3-fold) without detectable off-target effects, including at the adjacent *F10* gene. Overall, these data demonstrated for the first time the ability of a single TALE-TF module to rescue transcription impaired by promoter mutations.

Very recently, a CRISPR activation (CRISPRa) system based on deactivated Cas9, fused with a tripartite transcriptional activator (VPR) and driven to the target by a single guide (sg)RNA, has been described for the transactivation of *F7* and *F8* promoters [67]. In particular, luciferase-based assays in hepatoma cells identified sgRNAs able to significantly increase the activity of either wild-type or severely defective (c.-61T>G mutation) *F7* promoter and to act on the endogenous *F7* gene by promoting FVII secretion/activity, as well as to transactivate the *F8* promoter in hepatic and endothelial cell lines, the latter more physiological for FVIII. It is worth noting that, at variance from the typical CRISPR application, the CRISPRa system does not involve the cleavage of the target sequence but rather its recognition, which should reduce detrimental off-target effects. Moreover, these specific features of the CRISPRa system suggest that the targeting of sequences outside those involved in transcriptional control would be ineffective, which would further restrict the number of potential off targets.

Overall, genome engineering is generally considered to have various advantages over viral vector-based gene therapy, including the possibility to precisely edit the target loci, decreased insertional mutagenesis and maintain the physiological transcription (endogenous promoter) of the edited locus. However, despite the therapeutic potential of genome editing, its translatability in vivo still requires viral vectors (i.e, AAV) to deliver the nuclease and donor DNA, falling back to the same AAV gene therapy limitations, including the constraints of cassette size and immunological concerns. Although the delivery of mRNA encoding the nucleases can circumvent some of the vector-based gene therapy limitations, the main limitation of genome engineering relies on the general low efficiency and the intrinsic risk of off-target effects.

## 4. Modulation of Splicing for Therapeutic Purposes

### 4.1. The Splicing Process

**Figure 4:**
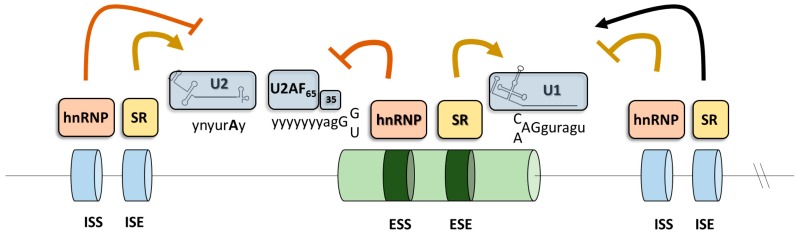
Elements regulating the splicing of pre-messenger RNA (mRNA). Major regulatory elements of pre-mRNA splicing, namely donor/acceptor splice sites and polypyrimidine tract, are shown with the corresponding trans-acting factors (U1, U2 snRNPs and U2AF_65_-_35_). The Exonic Splicing Enhancer (ESE), Silencer (ESS), Intron Splicing Enhancer (ISE) or Intron Splicing Silencer (ISS) sequence(s) positively or negatively contribute to exon recognition through interaction with serine–arginine-rich (SR) proteins and heterogeneous ribonuclear particles (hnRNPs), respectively.

The process called RNA splicing is required to produce mature mRNA encoding proteins. In fact, small coding segments (exons) scattered across the whole genome carry the information for the synthesis of proteins and represent no more than 1% of the entire genetic information [68]. Therefore, the intragenic regions (introns), the segment of DNA that is located between two exons of a gene, have to be removed. Thus, RNA splicing is similar to an audiotape, where the unwanted pieces are clipped out and then taped together to produce the desired sound. Because of the triplet nature of the genetic code, a mistake in cutting the RNA of only one nucleotide will produce an mRNA with an altered reading frame, thus producing a message that cannot encode the correct protein. Therefore, the splicing process must be very precise. In introns, the 5’ donor splice site (5’ss), the branch site (near the 3’ end of the intron) and the 3’ acceptor splice site (3’ss) are required for intron removal and these elements are respectively recognized in the early step of spliceosome assembly by the U1snRNP, U2snRNP, and U2-auxiliary factors (U2AF35 and U2AF65) heterodimer. While the chemistry reaction of splicing is simple, the exact identification of exons in very long introns is a complicated task, not yet really understood. Therefore, there are additional splicing regulatory elements involved in exon/intron boundary identification (Figure 4). According to their location and activity, the auxiliary elements are known to function as exon splicing enhancer and silencers (ESEs and ESEs, respectively) or intron splicing silencers (ISSs and ISSs, respectively). Typically, serine/arginine-rich (SR) proteins recognize enhancer elements whereas heterogenous nuclear ribonucleoproteins (hnRNP) interact with silencer elements, thus promoting or impairing exon definition, respectively [69].

In many cases, the splicing process can create many protein isoforms by varying the exon composition of the same mRNA. This phenomenon is called alternative splicing and allows the cell to expand the diversity of proteins from a relatively small number of genes. It has been calculated that more than 90% of human genes undergo alternative splicing, thus providing extensive opportunities for gene regulation during development, cell differentiation, and homeostasis [70,71,72].

An extra layer of complexity is related to the interplay among splicing, transcription and chromatin remodeling processes. In fact, splicing factors are recruited on nascent pre-mRNA by the RNA polymerase through its carboxy-terminal domain. Moreover, the elongation speed of RNA polymerase provides a kinetic window for the assembly of splicing factors as well as for the remodeling of RNA folding structure, thus influencing the splice site selection and therefore the exon fate [73,74,75]. In this view, post-translational modifications of histones, chromatin remodeling factors and nucleosome position can directly influence the speed of transcription as well as the recruitment of adapters that interact with splicing regulators. All these aspects have been demonstrated in several studies, demonstrating their crucial role in modulating the RNA splicing outcome [69,76,77,78,79].

### 4.2. Altered Splicing in Human Diseases

The selection of the proper 5’ and 3’ss is complicated by the presence of multiple cryptic donor and acceptor splice sites throughout the genome. Moreover, since the splicing process is associated with RNA transcription, nuclear RNA exportation as well as chromatin remodeling, multiple regulatory machineries must properly interface to ensure correct splice site selection [80]. Because of all these players and the number of proteins involved, it is not surprising that point mutations affecting one of these processes are associated with impaired pre-mRNA splicing and are often associated with human diseases (inherited or acquired) [81].

Generally, splicing mutations are considered as all those nucleotide variants located within the common and conserved splicing regulatory elements, more precisely the 5’ss, 3’ss, and the branch point site. These nucleotide changes generally impair splicing by destroying (or weakening) the affected splice site or by creating (or strengthening) cryptic splice sites, thus leading to the production of aberrant mRNAs (exon skipping, partial or full intron inclusion) that undergo nonsense-mediated decay (NMD) or encoding defective proteins. Moreover, also nucleotides changes impairing the expression of splicing factors are generally associated with human diseases [82,83,84]. It is also worth noting that since the amino acid code within exons overlaps with the splicing code consisting of ESEs and ESSs, also mutations within exons, either missense, nonsense or synonymous, can exert their pathogenic role by altering splicing, as it has been demonstrated in hemophilia B [85,86] and A [87]. Despite the occurring pathogenic aberrant splicing mechanisms, some traces of functional mRNA might be produced, leading to hardly detectable levels of functional proteins. While this aspect could not ameliorate the patients’ phenotype, it is crucial for all human diseases in which null protein function is incompatible with patients’ birth and survival, as demonstrated in the coagulation FVII deficiency [88].

The pathogenicity of new genomic variants is hardly predictable because of the degenerate nature of splice site consensus sequences. While nucleotide changes destroying the invariant GT or AG dinucleotides on 5’ss and 3’ss are always associated with aberrant splicing, the presence of a genetic variant on other positions is not always indicative of pathogenicity. Various in-silico prediction tools have been developed with the aim of predicting the effect of nucleotide changes on splicing, but this goal still seems far away [89].

### 4.3. U1 snRNP

The binding of the U1snRNP to the 5’ss of an intron is the first step of spliceosome assembly. The U1snRNP is composed of a 165-bp long small RNA (U1snRNA) complexed with seven Smith antigen (Sm) proteins, and three U1-specific proteins (U1-A, U1-70K and U1-C). In the U1snRNP, the 5’ tail of the U1snRNA directly interact 5’ss by complementarity [90].

The 5’ss motif is represented by the last three exonic and the first six intronic nucleotides, even if recent findings have demonstrated that even the seventh and eighth nucleotides in the intron (positions +7 and +8), although not conserved, can contribute to 5’ss selection in mammals [91], thus establishing up to 11 base pairs with the U1snRNA. Despite the conservation of the 5’ss sequence (consensus motif CAG/GURAGU, where R is a purine), in the human transcriptome there are reported more than 9000 5’ss variants, including a small proportion (~0.56%) in which a cytosine, instead of thymine, is located at position +2. The observation that mismatches between 5’ss and U1 tail are allowed poses the question how a single U1snRNP can mediate the recognition of a wide diversity of sequences. Recently, it has been demonstrated that the U1snRNA can recognized non-canonical 5’ss through new registers (alternative complementarity) [92] and indicating that recognition of 5’ss is a process far from being well understood. Considering the complexity and factors involved in 5’ss selection, the effects of nucleotide changes within the 5’ss may be difficult to predict without splicing pattern analysis.

### 4.4. Compensatory U1 snRNA to Rescue Splicing

In coagulation factor genes, nucleotide changes occurring at the donor splice sequences occur at a rate (8% in *F7*; 10% in *F9*; 3% in *F8*) (http://www.hgmd.cf.ac.uk/ac/index.php) very close to that (~10%) reported for other human inherited diseases [84,93]. These variants, by weakening the complementarity of the U1snRNA tail to the 5’ss, can result in aberrant splicing and reduced protein levels secreted in plasma, thus leading to hemorrhagic phenotypes.

The finding that compensatory U1snRNA can modulate splicing by promoting exon definition by 5’ss utilization came from the early attempts to prove that U1snRNA has to base pair to 5’ss to mediate the splicing of pre-mRNA [94]. Since that time, increasing evidences have showed that splicing defects caused by splicing mutations can be rescued with variants of U1snRNA, modified to restore the complementarity to the mutated 5’ss as well as to redirect the spliceosome machinery to proper exon-intron junction [86,95,96,97,98,99,100,101,102].

**Figure 5:**
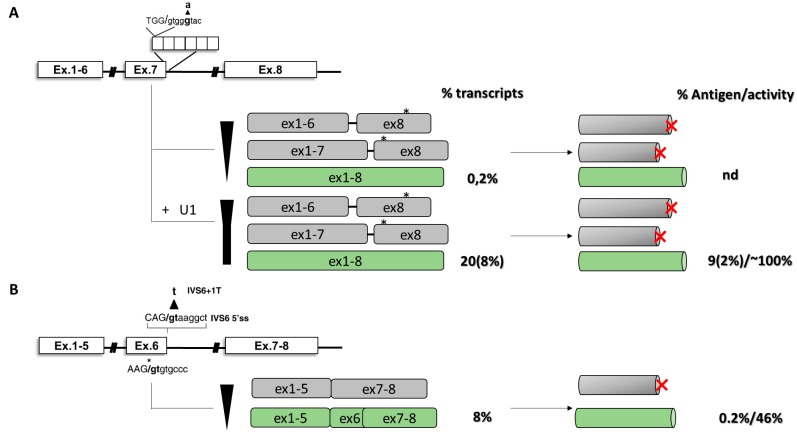
U1-mediated rescue in coagulation FVII deficiency. Coagulation FVII deficiency caused by c.859+5G>A (**A**) and c.681+1G>T (**B**) nucleotide changes in the *F7* gene. Schematic representation of the genomic context (left panel), splicing transcripts (middle panel) and protein isoforms (right panel) is reported. Sequences of the splice sites and position of mutations are indicated. Frameshift of the coding sequence and premature stop codons are reported by asterisks and red X letter, respectively. Percentages of transcripts, antigen and relative coagulation activity are reported. Values in rounded brackets indicate experiments in mouse model of the disease.

In coagulation FVII deficiency, the c.859+5G>A variant occurs at the + 5 position in the 5’ss of intron 7 (IVS7) (Figure 5A). The mutation is located in the first of a variable number of highly homologous 37-bp repeats, where an oriented scanning mechanism leads to the utilization of only the most upstream 5’ss in normal condition [103]. Homozygous patients for the c.859+5G>A nucleotide change experience life-threatening hemorrhagic symptoms and require replacement therapy. In cellular models, the c.859+5G>A results in exon skipping and the activation of intronic downstream cryptic 5’ss. Both transcripts, if translated, do not encode for functional proteins. The observed aberrant splicing induced by the mutation can be explained by the reduced complementarity, as well as inefficient recognition, between the mutated IVS7 5’ss and the 5’ tail of the U1-snRNA, thus reducing the overall exon definition. Interestingly, the co-expression of a compensatory U1 snRNA (U1+5A), designed to restore complementarity to the defective 5’ss, significantly rescued the splicing defect, leading to the synthesis of normal transcripts (Figure 5A) [98] and functional FVII molecules, the coagulant activity levels of which reached 10% of those of the normal construct, an extent that would be theoretically sufficient to correct the coagulation defect in patients [97]. Based on these encouraging results, the U1-mediated rescue of FVII biosynthesis was assessed in mice. Here, the U1-mediated rescue was clear and sustained for up to 8 weeks post-injection [104]. Although the limitations of mouse model of splicing FVII deficiency, the increase of circulating human FVII levels were modest and they could improve the coagulation phenotype if translated into patients. Overall, these data provided the first proof-of-concept that engineered U1snRNAs can efficiently rescue, at RNA and protein levels, in a mouse model splicing defects caused by splicing variants with increased circulating levels of a therapeutic protein.

Despite these encouraging results, not all splicing mutations can be efficiently rescued by modified U1snRNA-approach. In the coagulation FVII context, the *F7* c.681+1G>T mutation (IVS6+1) has been found in two homozygous FVII-deficient patients with life-threatening bleeding symptoms. Due to the highly conserved consensus sequence at the intronic +1 position of 5’ss, nucleotide changes at this position, a frequent cause of severe human genetic diseases [81], are also generally considered null mutations as they are predicted to completely disrupt the splicing process. Regarding the *F7* gene, the fatal perinatal bleeding in *F7* knock-out mice and the absence of homozygotes for large *F7* gene deletions indicate that minimal levels of FVII are essential for life, and suggest that trace levels of functional FVII should be produced even in the presence of the “null” mutation at the 5’ss. In vitro, it has been demonstrated that the c.681+1G>T, because of the presence of an in-frame cryptic exonic 5’ss, abrogates correct splicing but also induces the synthesis of an in-frame alternative *F7* transcript encoding a protein scarcely secreted but possessing a remarkable catalytic activity (Figure 5B) [88], thus ensuring a minimal hemostasis and reverting an otherwise perinatally lethal genetic condition. In this context, a strategy based on compensatory U1 failed to rescue coagulation FVII expression, highlighting the importance of position +1 for proper exon definition.

### 4.5. Exon-Specific U1 snRNAs (ExSpeU1)

The modified U1snRNA approach has the potential to affect the splicing of other genes (off-target effects), a drawback shared with other strategies targeting the RNA, and can rescue a single nucleotide change, thus limiting its therapeutic potential. In response to these limitations, new U1snRNA variants, named Exon-Specific U1 snRNAs (ExSpeU1), thanks to their ability to base pair with intronic and less-conserved sequences, have been developed. Their ability in rescuing splicing process have already been demonstrated for various diseases, in both in vitro and in vivo models [85,105,106,107,108,109,110,111]. It is worth nothing that the ExSpeU1snRNA-based approach is able to rescue different splicing mutations with one single engineered molecule, thus expanding its therapeutic translatability.

**Figure 6:**
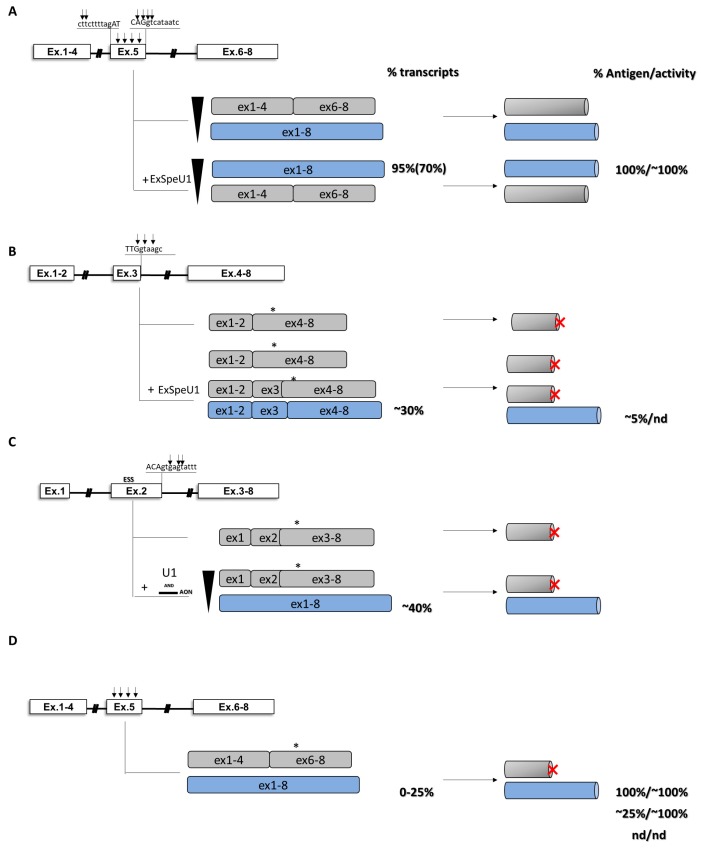
U1-mediated rescue in hemophilia B. Hemophilia B models caused by multiple nucleotide changes at the 3’ss (exon 5, **A**), at the 5’ss (exons 2, 3 and 5, **B**, **C** and **D**) or within exon (exon 5, panel **D**) of various exons of the *F9* gene. Schematic representation of the genomic context (**left** panel), splicing transcripts (**middle** panel) and protein isoforms (**right** panel) is reported. Sequences of the splice sites and position of mutations (black arrows) are indicated. Frameshift of the coding sequence and premature stop codons are reported by asterisks and red X letter, respectively. Percentages of transcripts, antigen and relative coagulation activity are reported. Values in rounded brackets indicate experiments in mouse model of the disease.

In the coagulation field, the ability of ExSpeU1 in restoring proper exon definition has been initially evaluated in the context of exon 5 of coagulation FIX [112]. In cellular models, variants located within the 5’ss (c.519A>C/G/T, c.520G>A/T, c.520+1G>A, c.520+2T>C) and the 3’ss (c.392-8T>G, c.392-9T>G) abrogated correct splicing of exon 5 and led to exon skipping. Here, a U1snRNA modified to restore complementarity with the mutated 5’ss was able to rescue nucleotide changes c.519A>C/G/T (Figure 6A). On the other hand, by using the c.519A>C as reference, a screening of ExSpeU1 snRNAs targeting intronic nucleotides from position 27 to position 63 revealed that all ExSpeU1 promoted exon definition, as observed by the reduction in exon 5 skipping and the appearance of correct transcripts. In particular, some of them (fix 1, fix 9 and fix 10) showed the strongest effect with a nearly complete rescue of aberrant splicing. To evaluate their ability in rescuing FIX expression, a full-length splicing-competent gene construct, in which the introns surrounding exon 5 were placed into the *F9* cDNA, was exploited. In cellular models, co-transfection of ExSpeU1 fix 9 resulted in a complete rescue of exon 5 inclusion for multiple mutations located in the 5’ss and in the 3’ss of exon 5 [112]. The rescue was clear at both RNA and protein levels, with the appearance of the full-length protein form showing activity levels comparable to those observed in the normal condition. If translated to patients, the correction would produce a therapeutic correction of the bleeding defect. Based on these promising results, the efficacy of ExSpeU1 fix 9 in rescuing splicing has also been demonstrated in a mouse model, where the correction was demonstrated at both RNA and protein levels in murine plasma [113].

The ability of engineered U1snRNAs to rescue splicing has also been proven in several human disease models [85,105,106,107,108,109,110,111,114,115], but nucleotide changes at the conserved GT nucleotide of 5’ splice sites (5’ss), frequent and associated with severe phenotypes, are thought to be not rescuable due to their conservation within the donor splice site. Recently, the ability of ExSpeU1 in rescuing nucleotide changes in the 5’ss of exon 3 of coagulation FIX, including some occurring at the highly conserve nucleotide positions +1 and +2, has been investigated the. In this context, an ExSpeU1snRNA demonstrated its ability in restoring proper exon definition in the presence of multiple nucleotide changes and, for the first time, also for a variant (c.277+2T>C) located in the highly conserved position +2. The rescue was evident at both RNA (~21%) and protein (~4%) levels (Figure 6B) [116].

Changes altering the conserved 5′ss GT dinucleotide have been thought to be essential for the correct splicing of pre-mRNA [117] and indeed not rescuable. Interestingly, a small proportion (~0.56%) of introns has a variant of the 5′ss containing a cytosine, instead of thymine, in position +2 [118] that is efficiently recognized by the U2-type spliceosome through the presence of strong consensus sequences maximized for base-pair formation with U1 and U5/U6 snRNAs. Overall, these data, together with this observation, support a mechanism in which nucleotide changes at +2, depending on the specific exon context, could still be recognized and rescued by U1snRNA, thus expanding the therapeutic potential of this approach.

### 4.6. Combination of Modified U1snRNAs and Antisense Oligonucleotides

The ability of ExSpeU1 in restoring proper exon definition, impaired by splicing variants, has also been evaluated in the exon 2 context of coagulation FIX. Here, the specific exonic context provided a new insight about a model in which the interaction of positive and negative regulatory elements leads to severe hemophilia B (Figure 6C) [86]. An analysis of the splicing pattern of exon 2 in human liver samples showed that, in physiological condition, a cryptic 5′ss located 104 bp upstream of the authentic 5′ss is used in ~20% of transcripts, leading to the formation of a deleted and frame-shifted mRNAs with a premature stop codon at position c.151. In cellular models, the expression of different variants (c.252+3G>C, c.252 + 5G>A, c.252 + 5G>C, c.252 + 5G>T and c.252+6T>C) located in the exon 2 5’ss showed that all nucleotide changes lead to the virtually exclusive usage of the exonic cryptic 5′ss and synthesis of aberrant transcripts. In normal condition, the presence of an adjacent exonic ESS conserved among species would limit the impact of the cryptic 5′ss on the expression of functional FIX protein by reducing the efficiency of unproductive splicing. In this context, an analysis of splicing revealed that all missense changes occurring within this ESS favor the cryptic 5′ss recognition and significantly decrease the levels of correct transcripts, suggesting that their detrimental impact on FIX expression could be attributable to a combination of reduced amounts of correct *F9* mRNA (∼40%) with the alterations produced by amino acid substitutions. In the attempt to rescue the splicing process, only a tailored approach based on the usage of an AON, designed to mask the cryptic 5′ss, and a modified U1snRNA, designed to restore proper exon definition, was able to significantly increase the selection of the authentic 5′ss and rescue splicing (from 0 up to 40% of correct transcripts) in the presence of all, but one, variants. Overall, these data provide new insights into the combinatorial activity of antisense oligonucleotides and compensatory U1snRNA in inducing splicing correction.

### 4.7. Missense Mutations and Altered Splicing

Missense changes are generally thought to elicit their pathogenic effect by altering protein function. However, since the splicing code overlaps with the amino acid code, particular attention has to be given to missense variants, with their involvement in splicing alteration being largely underestimated. It is worth noting that, albeit exonic variants are strong candidates to affect splicing, the relative contribution of splicing alteration and impaired protein function in the disease pathogenesis is largely unknown. The dissection of these splicing regulatory elements, as well as the understanding of the mechanisms underlying proper exon definition, is crucial to improve knowledge of splicing process, but it provides also new opportunities for splicing correction.

In a coagulation *F9* exon 5 context, the ability of engineered U1snRNA in rescuing splicing has also been tested for missense changes altering the pre-mRNA processing (Figure 6D) [105]. In this context, the splicing analysis of exonic changes (two synonymous, 11 missense and three nonsense mutations) occurring within various exonic regulatory elements mediating proper exon 5 inclusion revealed that nine out 16 exonic variants induced significant exon 5 skipping, while all of them impaired FIX activity at variable extents. Notably, an ExSpeU1, targeting intronic regions downstream the exon 5 5’ss, completely rescued all exon skipping events. Taken together, considering the previously reported five variants (Figure 6A) [112], a unique ExSpeU1 demonstrated its ability in rescuing 14 different changes that, with different mechanisms involved, share the exon skipping event of exon 5, increasing therefore the number of affected individuals that would benefit from this therapeutic strategy.

**Figure 7:**
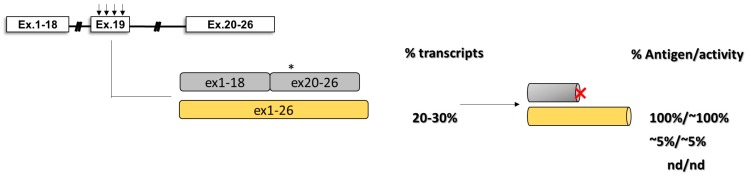
U1-mediated rescue in hemophilia A. Hemophilia A model caused by multiple nucleotide changes within the *F8* exon 19. Schematic representation of the genomic context (**left** panel), splicing transcripts (**middle** panel) and protein isoforms (**right** panel) is reported. Frameshift of the coding sequence and premature stop codons are reported by asterisks and red X letter, respectively. Percentages of transcripts, antigen and relative coagulation activity are shown on the right.

The impact of missense changes on splicing has also been investigated in hemophilia A, particularly for some nucleotide variants affecting the exon 19 context of coagulation FVIII (Figure 7) [87]. Here, by evaluating the splicing pattern in vitro and in ectopic mRNA from patients’ blood samples as well as the cofactor activity, it has been clearly demonstrated the interplay between differentially altered mRNA and protein patterns that, only in combination, account for moderate/severe hemophilia A phenotypes observed in patients.

### 4.8. Antisense Approaches for Splicing Modulation

Generally, the loss of one single exon has a tremendous impact on protein structure/function, and thus on patients’ phenotype. However, particularly for large genes, the modularity of protein, in which each exon generally encodes for a particular protein domain, offers the possibility to have shorter isoforms with reduced, but still active proteins. In this context, a strategy able to induce exon skipping through masking of splicing elements (5 and 3’ss) required to define the target exon would represent a valid therapeutic option. On the other hand, the presence of nucleotide changes, both intronic and exonic, leading to the activation of a cryptic splice site, or favoring the recruitment of splicing factors impairing the physiological splicing outcome opens the possibility to exploit antisense molecules with therapeutic purposes. Among different strategies, the usage of antisense oligonucleotides and engineered U7snRNA represents two valuable option to splicing modulation for therapeutic purposes.

In general, the usage of strategies based on antisense molecules for splicing correction and demonstrating their therapeutic potential in inherited diseases other than coagulation disorders has been illustrated in previous excellent reviews [45,46,48] and will not be described here.

In the coagulation *F9* exon 2 context, a cryptic 5′ss located 104 bp upstream of the authentic 5′ss is exclusively used in the presence of nucleotide changes are locate within the 5’ss and the identified ESS, respectively. In the attempt to rescue splicing, an engineered U1snRNA designed to restore proper exon definition, or an AON able to mask the cryptic 5’ss failed in rescue splicing. Only the co-delivery of both molecules resulted in partial correction of splicing [86] (Figure 6C).

The ability of AON in restoring proper mRNA processing has also been provided in the model of coagulation FV deficiency. In this context, the research of causative mutations in patients revealed the presence of a synonymous variant in exon 8 (c.1281C>G) or of a deep-intronic mutation (*F5* c.1296+268A>G), both of them activating a cryptic 5’ss and leading to the formation of a pseudo-exon [119,120]. In both contexts, the delivery of AON, morpholino oligonucleotide or of an engineered U7 small nuclear RNA, all of them designed to mask the cryptic 5’ss, demonstrated their efficacy in restoring proper mRNA processing in a dose-dependent manner. Significantly, the therapeutic approach has been demonstrated in vitro and, for the *F5* c.1296+268A>G variant, also in the patient’s megakaryocytes ex vivo.

Another interesting example on how antisense molecules can represent a valid option in rescuing splicing in pathological condition is represented by the model of afibrinogenemia caused by a homozygote deep intronic mutation (FGB c.115–600A>G) [121]. In this context, the mutation is associated with the inclusion of a cryptic exon through the creation of a new ESE motif recognized by splicing factors. A morpholino oligonucleotide able to impair recognition of the cryptic ESE demonstrated its ability in rescuing correct splicing which efficacy accounted for >50%.

### 4.9. Intervention at the RNA Level by siRNAs

The outcome of alternative splicing in pathological conditions can generate disease-specific transcripts that represent the ideal target for gene silencing tools. This is particularly true when the new messenger encodes for a protein with a dominant negative activity. In this context, allele-specific silencing can represent a valid therapeutic option, as demonstrated by the commercialization of various drugs exploiting the RNA interference (RNAi) mechanism. RNAi can be achieved by different strategies, and exploitation of short interference RNA molecules (siRNA) is by far the most commonly used approach to induce it. In coagulation disorders, the von Willebrand disease represents a paradigmatic example of dominant negative manifestation of a disease, where in some heterozygous patients the new protein is able to impair the VWF dimerization and multimerization, thus providing the rationale for the dominant inheritance. In this context, exploitation of siRNA approach demonstrated its ability in inducing the allele-specific silencing, thus improving VWF function. This approach has been evaluated for single-nucleotide polymorphisms (SNPs), a missense (p.Cys2773Ser) and an in-frame deletion (p.P1127_C1948delinsR) changes [49,122].

Outstanding results exploiting the siRNA have been reported for antithrombin, a potent inhibitor of thrombin generation. Here, in the attempt to promote hemostasis in hemophilic patients by favoring the pro-thrombotic pathway of coagulation, the researchers developed a siRNA (Fitusiran) able to significantly (<50%) reduce antithrombin levels. The therapeutic potential, as demonstrated by increased thrombin generation, has been demonstrated in both animal models and patients with hemophilia A and B [123,124].

Among therapeutic strategies tailored on molecular mechanisms, intervention at the RNA level has different strengths. In fact, RNA targeting would allow the restoration of gene expression while maintaining the physiologic gene regulation. Since the presence of the transcribed RNA, both mature (mRNA) or its precursor (pre-mRNA), is essential, it is clear that therapeutics acting at the RNA level can exert their effect only in physiological tissue of gene expression, thus limiting the off-target effect due to expression in other tissues. Moreover, since the coding cassette of RNA targeting molecules is generally limited, virtually any virus-mediated delivery approach can be exploited, thus expanding the available options. However, as any therapeutic approach, intervention at the RNA level could induce off-target effects by intervening on other RNA substrates and any effort has to be made to reduce them. Moreover, strategies to deliver RNA targeting molecules relies in the category of approaches for gene therapy, thus sharing the same immunological concerns (presence of neutralizing antibodies toward the viral capsid) and vector dilution effect (loss of expression in infants).

## 5. Rescue of Coagulation Factor Expression by Translational and Post-Translational Modulation

Protein synthesis is a high-fidelity process involving different effectors and the interplay of which leads to the synthesis of new polypeptides through the ribosome-mediated translation of mRNA transcripts [125,126].

Nonsense and missense mutations, which overall represent the most frequent gene alterations arising from point mutations, may affect protein synthesis at translational or post-translational levels or both. In general, nonsense mutations may result in the production of potentially unstable truncated proteins with loss-of-function features, whereas missense mutations may exert pleiotropic effects on protein biosynthesis/processing and/or secretion/function as well as affect pre-mRNA splicing when falling into exonic sequences, as exemplified by the several variants characterized in the coagulation field [105,110,127,128,129,130].

Noticeably, knowledge of the effects of these mutation types on protein synthesis has led to the exploration of approaches aimed to overcome premature termination or to correct protein folding defects with the potential rescue of protein synthesis/function as the final goal.

### 5.1. Translation Termination, Nonsense Mutations and Ribosome Readthrough

**Figure 8:**
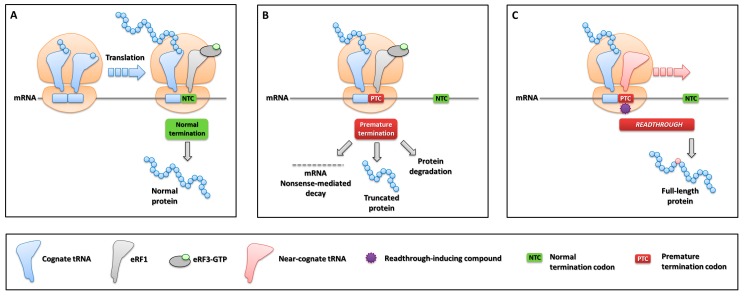
Translation termination and readthrough-mediated PTC suppression. (**A**) Normal translation termination involving eRF1 and eRF3-GTP at 3’ natural stop codons. (**B**) Aberrant translation termination at PTCs and possible outputs resulting from nonsense changes. (**C**) Mechanism of ribosome readthrough resulting in PTC suppression, either spontaneous or induced by compounds, and synthesis of the full-length protein.

An essential determinant for translation, together with the start codon AUG (coding for methionine), is represented by termination signals (UGA, UAG, UAA stop codons), which are normally located at the end of coding regions. When the A-site of translating ribosomes is occupied by one of the three stop codons, the recruitment of the releasing factor eRF1 elicits the termination of protein synthesis and the release of the nascent polypeptide [131] (Figure 8A). However, translation termination may become a pathological event when a nucleotide change causes the modification of a sense codon to a premature termination codon (PTC), abnormally located upstream of the natural termination signal, resulting in the so-called nonsense mutations. A meta-analysis has revealed that this mutation type represents ~11% of all gene lesions causing human inherited diseases [132].

The outcome of genetic disorders caused by nonsense mutations is modulated by a series of molecular events strongly impairing gene expression, particularly in homozygous or hemizygous conditions, and contributing to shape the so-called “null” phenotypes. In particular, a PTC would result in (i) the down-regulation of aberrant mRNAs through the mRNA NMD surveillance mechanism, the efficiency of which depends on the position of PTCs [133,134] and/or (ii) the degradation of misfolded/unfolded truncated proteins arising from premature translation termination (Figure 8B).

Despite the high fidelity of protein synthesis, translation termination elicited by a PTC is not 100% efficient. Indeed, at very low rates in normal conditions (~10^-4^), a process called ribosome readthrough may lead to the synthesis of full-length proteins through mis-recognition of the PTC and incorporation of an amino acid at the nonsense position [135]. This particular event is driven by near-cognate tRNAs that, having anticodons complementary to two out of three positions of a PTC, outcompetes eRF1 at the ribosome A-site (Figure 8C). Interestingly, a recent study has indicated that mispairing at stop codon position 1 or 3 mediates the interaction between PTC and a near-cognate tRNA anticodon [136].

The efficiency of readthrough is influenced by the PTC sequence context, namely the type of stop codon and the downstream nucleotide (referred also as tetranucleotide [137]). Manuvakhova and co-workers evaluated the occurrence of readthrough by in vitro studies with constructs bearing all possible combinations of tetranucleotides, and demonstrated a differential degree of suppression depending on the stop codon type (UGA≥UAG>UAA) and downstream nucleotides (C>A>G,U), with UGAC being the most readthrough-prone sequence context [138].

Ribosome readthrough, which belongs to the so-called “recoding” processes [139], is not only a mechanism that may overcome aberrant translation termination, but also plays a role in modulating gene expression through selective suppression of stop codons. Indeed, readthrough is essential for the expansion of small genomes, such as those of viruses, by producing different effector proteins through targeted suppression of PTCs in overlapping coding sequences [140]. On the other hand, it has been described as the process underlying the production of selenoproteins through insertion of selenocysteine, also called as the 21^st^ amino acid [141], over natural UGA stop codons characterized by a downstream hairpin structure called selenocysteine inserting sequence [142]. Finally, regulated suppression of UAG stop codons leads to the insertion of pirrolysine (the 22^nd^ amino acid) [143,144]. Overall, sequence determinants represent hallmarks for PTCs susceptibility to suppression, thus indicating that the occurrence of readthrough is related to the “leakiness” of termination signals.

The evidence that some drugs are able to alter translation fidelity by acting on ribosome function has revealed the potential implications of this side-effect in terms of therapeutic approaches for genetic disorders caused by nonsense mutations [145]. In particular, antibiotic drugs such as aminoglycosides alter the proof-reading capacity of ribosomes by binding to the decoding center located in the small subunit [146]. The direct consequence at a nonsense codon is the mis-incorporation of amino acids rather than of releasing factors. This results in a frequency of translational readthrough higher than that of polypeptide chain termination, thus leading to the synthesis of full-length proteins (Figure 8C). Since the first description of drug-induced readthrough as a potential therapy for nonsense mutations in the *CFTR* gene [147], several studies have investigated the effectiveness of PTC suppression in vitro and in vivo for genetic disorders, thus opening the consideration of this therapeutic approach as a form of personalized medicine. Several disease models have been successfully challenged with the drug-induced readthrough approach, including Duchenne/Becker muscular dystrophies, cystic fibrosis, spinal muscular atrophy, and lysosomal storage disorders [148,149].

**Figure 9:**
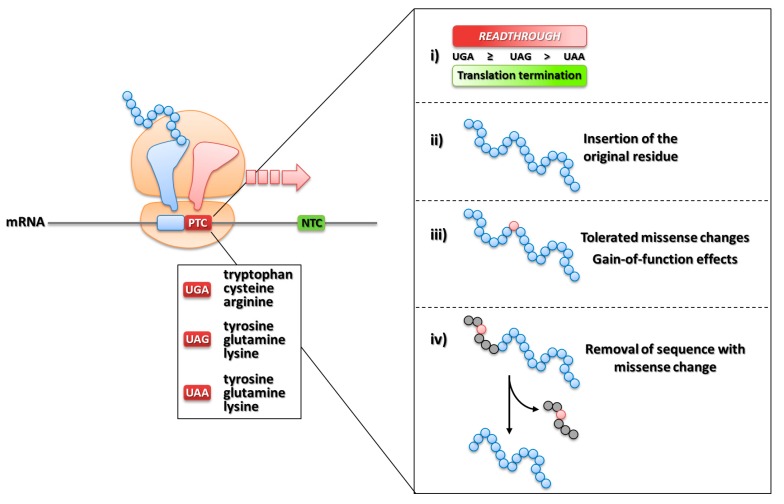
Amino acid insertions and productive protein outputs arising from readthrough. Type of amino acids inserted at PTCs as a function of the type of stop codon (**left** panel) and molecular determinants influencing the productive output of readthrough (**right** panel).

The amino acid inserted at the PTC position could be different than that encoded by the wild-type codon, an event that might counteract or mitigate the beneficial impact of readthrough in terms of protein structure/function. Interestingly, recent studies have identified differential incorporation rates as well as types of amino acids inserted over a PTC (Figure 9, left panel), namely tryptophan/cysteine/arginine at UGA and glutamine/tyrosine/lysine at UAG/UAA [150,151]. These data may help interpreting the impact of readthrough from the protein point of view, with potential implications for the identification of eligible nonsense mutations, which is particularly relevant for proteins with enzymatic activity.

In this scenario, the occurrence of spontaneous as well as drug-induced readthrough appears relevant, in particular in homozygous (autosomal) and hemizygous (X-linked) disease conditions, with the important implication of partially restoring a protein product encoded by a unique copy of the affected gene.

### 5.2. Nonsense Mutations in Coagulation Factor Genes

Nonsense mutations are relatively frequent in coagulation factor disorders. In particular, in the mutational pattern of the X-linked hemophilia A and B, nucleotide changes resulting in nonsense mutations are approximately 10–14% (http://www.factorviii-db.org; http://www.factorix.org) [3,5]. This mutation type has also been found, albeit with different frequencies, in severe disease forms in almost all other bleeding disorders causing defects in serine proteases, cofactors and inhibitors as well as proteins involved in primary hemostasis and clot formation/stabilization (http://www.hgmd.cf.ac.uk/ac/index.php).

In this scenario, noticeable exceptions exist, namely (i) the p.R462X nonsense mutation in the *F7* gene, found in unrelated homozygous patients, which produces very low levels of a 5-residue truncated gain-of-function FVII molecule associated with an asymptomatic phenotype [152,153], and (ii) the *F10* gene, in which nonsense mutations are extremely rare [154,155], with those occurring at the carboxyl-terminal region being predicted to be asymptomatic due to the slight impact on FX secretion/function [156].

### 5.3. Pioneer Studies on Readthrough in Bleeding Disorders

Although our focus here is on in vitro and in vivo studies on drug-induced readthrough and on the molecular determinants underlying responsiveness of nonsense mutations, it is worth to note that a study conducted in 2012 has provided the intriguing evidence on the occurrence of spontaneous readthrough in hemophilia B patients with frequent *F9* mutations [157]. Importantly, at variance from the majority of human diseases, in coagulation factor deficiencies very low levels of functional protein (in the range of 2–5%) have relevant pathophysiological implications. Indeed, even tiny increase in functional protein levels in plasma would significantly ameliorate the clinical phenotype of patients [37]. For this reason, the extent of functional rescue promoted by readthrough is compatible with the low therapeutic threshold of bleeding disorders. The main findings obtained in the studies described below are summarized in Table 2.

A pioneer attempt of aminoglycoside treatment in hemophilia was conducted by James and co-workers in 2005 [158]. In this setting, gentamycin (7 mg/kg once a day) was administered to severe hemophilia A and B patients with PTCs in *F8* (p.R446X, p.S1414X, p.R2135X) and *F9* (p.R252X and p.R379X) genes. Treatment resulted in a transient shortening of coagulation times, namely the time needed to form a clot, and an increase in FVIII (1.6%) and FIX (2%) activity for the *F8* (p.S1414X) and *F9* (p.R379X) nonsense variants, respectively. However, in the remaining three patients (hemophilia A, p.R446X, p.R2135X; hemophilia B, p.R252X) the effect on functional parameters was not detected. The readthrough approach with aminoglycosides was also tried in vivo in a hemophilia B mouse model expressing the human FIX p.R75X and p.R384X nonsense variants [159]. Treatment with geneticin induced the increase (~5%) in FIX protein and activity levels in mice expressing the p.R384X variant but not in those harboring the p.R75X.

In rare bleeding disorders, such as FVII deficiency, nonsense mutations are very rare, particularly in the homozygous state (six reported, with the noticeable exception of the gain-of-function p.R462X variant [152]), and are mostly associated with severe phenotypes due to the pivotal role of FVII, the complete absence of which is virtually incompatible with life [9]. A first attempt on readthrough-mediated correction was explored on the p.K376X and p.W424X nonsense variants in cellular models [164] and patients [165]. Expression studies in mammalian cells revealed a dose-dependent rescue after treatment with geneticin and gentamycin, which however produced partially functional proteins with activity compatible with the low therapeutic threshold. The partial restoration of full-length FVII was also detected inside cells through fluorescence studies with FVII-GFP chimeric constructs bearing the two nonsense mutations [164]. Based on these results obtained in vitro, a pilot clinical study with gentamycin (3 mg/kg once a day) was conducted on severe FVII-deficient patients bearing the p.K376X and p.W424X nonsense variants. However, despite a slight shortening of coagulation times and the detection, through very sensitive fluorogenic assays, of a FVII activity above the baseline, treatment resulted in protein/activity levels on average undetectable [165]. Indeed, only sub-therapeutic FVII levels were observed in patients, probably due to minimal effects of gentamycin on FVII plasma levels, low suppression efficiency and/or amino acid insertions incompatible with a significant functional rescue.

### 5.4. Investigation of Readthrough Determinants in Bleeding Disorders

The interplay between favorable sequence and protein features to achieve a functional rescue by induced readthrough have been investigated in two different models of bleeding disorders (FVII deficiency and hemophilia B) by evaluating qualitative and quantitative readthrough components. Investigation of the molecular determinants underlying the interplay between nucleotide (mRNA) and protein (amino acid insertions) contexts contribute to shape the responsiveness of PTCs as well as the impact of readthrough on the resulting protein output.

A hypothesis-driven attempt was made for the homozygous p.S112X (UGA-A, <1% activity) [166] and p.C132X (UGA-C, ≥1% activity) [167] *F7* nonsense variants [163]. These mutations are associated with severe or unexpectedly moderate FVII deficiency, respectively, and are predicted to produce severely truncated FVII polypeptides devoid of the catalytic triad and thus inactive. As expected, in expression studies the induction of readthrough by geneticin increased protein and functional levels up to 3% (112X) and 13% (132X) of wild-type protein, which was compatible with the synthesis of full-length functional FVII proteins. Noticeably, low or very low FVII protein and activity levels were detected in basal conditions without the addition of geneticin, thus indicating the occurrence of spontaneous, albeit differential, functional suppression of the two nonsense mutations. In this view, the different phenotypes of patients might underlie the presence of traces of functional FVII arising from readthrough as a function of leakiness of PTC (UGA-C>>UGA-A) sequence contexts. Although re-introduction of the original residue might be hampered by the type of amino acid encoded by the wild-type triplet, tolerated amino acid changes due to permissive protein positions may result in functional full-length proteins. The protein output was further investigated by the expression of missense variants predicted to arise from readthrough over the UGA PTC [150], which (i) produced a moderate parallel decrease in secreted/functional FVII levels, thus indicating a less favorable sequence context in terms of suppression but a protein context tolerating missense changes (position 112), or (ii) completely abolished secretion and function, thus pointing toward that only the readthrough-driven incorporation of the wild-type cysteine is compatible with the observed functional readthrough (position 132). Overall, the degree of PTC suppression may shape the minimal functional threshold associated with spontaneous readthrough events, with important implications on phenotype severity.

The influence of molecular determinants have been deeply investigated in the model disease hemophilia B. Expression studies were conducted on an ample panel of PTCs (*n* = 11) representing the most frequent *F9* nonsense mutations, including all recurrent UGA PTCs at CpG sites, reported in ~70% (324 out of 469) patients with severe hemophilia B [161]. Among the UGA variants (*n* = 7), all predicted to be permissive in terms of readthrough [138], only the W240X (UGA-C) and R384X (UGA-U) were remarkably rescued in terms of FIX protein (W240X, ∼9% of wild-type; R384X, ∼2%) and activity (W240X, 5%; R384X, ∼8%). The specific features of the two nonsense variants help understanding the interplay between the two different components (nucleotide and protein contexts) driving responsiveness to readthrough. Noticeably, the relevant functional suppression observed for the W240X (UGA-C) is explained in light of the fact that re-insertion of the wild-type tryptophan is expected at this position [150]. The association with severe hemophilia B of the naturally-occurring missense changes at the 240 position (p.W240C [168] and p.W240R [169]), overlapping those introduced by readthrough other than tryptophan, confirmed that only re-introduction of the original residue was compatible with the observed functional output. Strikingly, activity of the R384X (UGA-U) after geneticin treatment was unexpectedly higher than the predicted susceptibility of its sequence context and, most importantly, of the observed protein levels, thus indicating that functional suppression at this position resulted in gain-of-function features. This finding was confirmed by the expression of the most frequent missense change (R384W) predicted for UGA PTCs, which revealed a similar hyperactive output. It is worth to note that the R384X PTC overlaps with the so-called “FIX Padua” position, in which the R384L substitution has been associated with a hyperactive FIX variant [162]. Overall, these observations indicated that nucleotide and protein constraints may limit the responsiveness of PTCs to readthrough, with a significant functional rescue requiring the combination of favorable mRNA sequence and protein features.

The impact of favorable features of PTCs was further detailed by investigation of three paradigmatic examples of nonsense variants (p.G21X, p.C28X and p.K45X) affecting the FIX signal and pro-peptide [160], two crucial regions for targeting to the endoplasmic reticulum [170] and for the major post-translational modification (γ-carboxylation) [171,172]. Geneticin treatment of cells expressing the G21X led to a significant parallel increase (∼4% of wild-type) in both secreted protein and coagulant activity levels over the baseline (∼0.4%). Strikingly, the specific coagulant activity, referred to as the activity/antigen ratio, of the G21X after treatment was compatible with normal FIX function, thus indicating a FIX protein with wild-type features. These observations were supported by expression studies with the predicted readthrough-deriving missense variants (G21W/R/C), which showed a preserved specific activity, thus demonstrating the production of FIX proteins with wild-type features upon readthrough and removal of the pre-peptide. On the other hand, response of the C28X and K45X variants to geneticin treatment was prevented by sequence constraints of adjacent (C28, between pre- and pro-peptide; K45, between pro-peptide and mature FIX) cleavage sites essential for FIX processing, a finding in line with the severe/moderate hemophilia B associated with missense mutations at these positions [173,174,175]. All these elements suggest that, for secreted proteins, the localization of PTCs in pre-peptide regions, which are intracellularly removed, would favor the synthesis and secretion of full-length wild-type proteins upon readthrough.

Finally, in a very recent study with reporter constructs, Liu and co-workers for the first time investigated the responsiveness of a panel of *F8* nonsense mutations with different sequence contexts. By combining expression studies with variants bearing PTCs as well as the predicted readthrough-deriving amino acid substitutions, mimicking the impact of missense changes on secretion/function of the resulting full-length proteins, a restricted number of potential candidates for therapy based on readthrough have been identified [176].

### 5.5. Molecular Determinants Underlying Productive Readthrough over PTCs

The data stemming from the works described above provide evidence that a successful functional suppression of nonsense mutations through drug-induced readthrough (Figure 9, right panel) can be achieved by the combination of:

(i) the degree of the susceptibility of sequence contexts to suppression, also promoted by the presence of readthrough-inducing compounds;
(ii) the insertion of the original residue permitting the synthesis of a wild-type full-length protein;
(iii) the insertion of amino acids tolerated for protein synthesis and function, or originating rare gain-of-function features providing advantageous protein outputs;
(iv) the favorable localization of nonsense mutations in protein sequences (i.e., signal peptides) that are removed during processing.

Overall, these findings indicate that, for proteins with enzymatic activity, the nucleotide context and protein features substantially restrict the number of PTCs compatible with functional rescue. This evidence provides the experimental bases to interpret the highly variable responsiveness of PTCs and could help (i) evaluating the features of PTCs in terms of nucleotide and protein sequence contexts/determinants, and (ii) predicting PTCs responsiveness in terms of suppression efficiency and protein output, the latter representing the driving force for the functional impact of the readthrough correction approach. The occurrence of NMD would introduce a further element of complexity to be considered due to its surveillance role and involvement in lowering the PTC-bearing transcripts potentially available for undergoing the readthrough process.

### 5.6. Protein Folding Defects and Chaperone-Like Compounds

**Figure 10:**
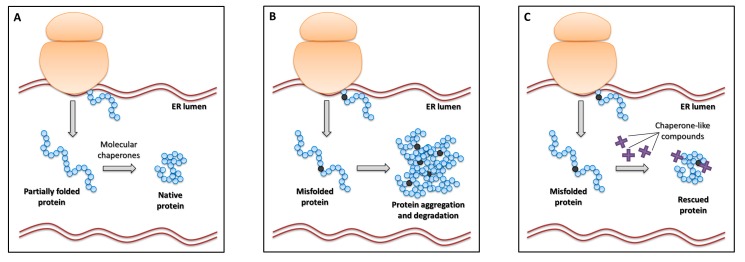
Impact of missense changes on protein folding and correction approaches through chaperone-like compounds. (**A**) Protein folding in normal conditions assisted by molecular chaperones, with a final production of the native protein conformation. (**B**) Aberrant folding and the consequent aggregation/degradation of misfolded proteins, unable to reach the native conformation, due to amino acid substitutions caused by missense changes. (**C**) Rescue of folding of misfolded proteins mediated by chaperone-like compounds.

Missense mutations are the main cause of human genetic diseases and the most detrimental effect is exerted by changes impairing protein folding. As a consequence, the altered protein is retained intracellularly or undergoes preferential degradation. The folding process is mediated by specialized molecules called molecular chaperones, which bind and stabilize nascent polypeptides, discriminating between folded and misfolded proteins and thus representing a key quality control mechanism [177,178]. On the other hand, polypeptides that fail to fold properly are degraded by proteasomes (Figure 10A,B) [179]. The accumulation of unfolded proteins may eventually lead to endoplasmic reticulum (ER) stress and the activation of the unfolded protein response [180].

Extensive investigations have revealed that several small molecules, named chemical/pharmacological chaperones, are able to modulate folding and rescue protein biosynthesis, with intriguing therapeutic implications (Figure 10C) [181].

Chemical chaperones are non-selective in their ability to stabilize mutant proteins and facilitate folding by supporting escape from ER quality control systems [182,183]. These molecules can be classified into hydrophobic compounds, such as dimethyl sulfoxide or sodium phenylbutyrate (Na-PBA), osmolytes such as polyols (glycerol, sorbitol), amino acids and derivatives (glycine, taurine), methylamines (betaine, trimethylamine *N*-oxide -TMAO-) and bile acids such as tauroursodeoxycholic (TUDCA) or ursodeoxycholic (UDCA) acids [182,184]. For hydrophobic chaperones such as Na-PBA, the interaction of hydrophobic regions of the compound with exposed hydrophobic regions of the target protein has been proposed as the possible mechanism of action [184]. On the other hand, osmolytes stabilize proteins by shifting the balance of native/denatured states towards the native state through minimization of the water-protein interface area [185]. In vitro studies have indicated that DMSO, glycerol, 4-PBA, TMAO sorbitol and myo-inositol are capable of restoring the expression of the cystic fibrosis transmembrane conductance regulator (CFTR) impaired by the frequent p.F508del mutation [186,187,188,189,190]. In addition, 4-PBA and glycerol have also been proven to efficiently rescue the secretion of α1-antitrypsin impaired by the common α1-AT Z variant, both in cellular and mouse models [191]. In addition, other molecules with unexpected chaperone-like effects have shown their potential as candidates for Gaucher (ambroxol hydrochloride, a commercially-available expectorant drug) or Pompe (*N*-acetylcysteine) diseases [192,193,194].

Pharmacological chaperones are low molecular weight compounds acting as folding correctors on a particular target to prevent faulty or to recover misfolded conformers, thus resulting effective drugs for protein rescue and/or protection from degradation [195]. Their action is mediated by the binding of proteins and the induction of refolding/stabilization, with the potential restoration of protein function [196]. These compounds have been successfully applied to enhance proteostasis of different protein types including transporters [197], aggregation-prone proteins [184,198] as well as lysosomal enzymes, G protein-coupled receptors, CFTR, with particular reference to the new VX-809 compound that has also entered clinical trials, as well as a wide range of human diseases [199,200,201].

With this as background, chemical/pharmacological chaperones are intriguing molecules for the development of innovative therapies for human genetic diseases, including bleeding disorders, caused by mutations impairing intracellular protein processing.

### 5.7. Intervention on Defective Protein Folding in Bleeding Disorders through Chaperone-Like Compounds

Coagulation factor deficiencies represent ideal models to evaluate the potential of alternative therapeutic approaches based on chaperone-like compounds. Indeed, missense mutations, and particularly those forms with a marked reduction in secreted protein levels, are a main cause of disease forms. To date, very few examples exist on the rescue of protein expression by chaperone-like compounds in coagulation factor deficiencies.

Two paradigmatic examples have been provided for in vivo models on the biosynthesis of FVIII and the associated deficiency hemophilia A. Malhotra and co-workers provided the first evidence on the use of a compound acting by alleviating ER stress, which represents a key event related to protein misfolding [180]. They showed that the lipid-soluble antioxidant butylated hydroxyanisole (BHA), a compound often added to foods to preserve fats, improved folding of FVIII, a molecule with a complex biosynthesis [90], and reduced ER stress as well as increased its secretion both in vitro and in vivo. In particular, BHA feeding reduced intracellular FVIII accumulation in mouse liver, with a consequent increased secretion of FVIII in plasma [202]. Interestingly, BHA also improved the secretion of the R593C variant, known to associated with hemophilia A due to a folding defect [203]. Interestingly, another mouse model of hemophilia A was challenged with the chemical chaperone betaine, frequently used as food supplement, which showed the ability to rescue FVIII folding and ameliorate the associated bleeding phenotype after oral administration [204]. In the same setting with BHA, the folding-defective mutant Q305P [205] was also rescued both in vitro and in vivo. Of notice, a BHA-specific output was observed as increased FVIII and FIX plasma levels in knockout mice after gene transfer, a finding that was further confirmed by the decrease in FVIII/FIX levels upon discontinuation of BHA treatment.

The approach with chaperone-like compounds such as Na-PBA was successfully tempted also to correct the folding/secretion of protein variants of coagulation vitamin K-dependent serine proteases such as protein C (PC) and FIX. In the first model, cells expressing the A267T PC variant with defective intracellular transport [206] were treated with several compounds, but only Na-PBA resulted in a dose-dependent (up to ~3 fold) and significant increase in PC antigen levels [207]. In the second model, Na-PBA appreciably improved, in a dose-dependent manner, the intracellular trafficking and secretion of the most frequent FIX missense variant p.R294Q associated with proportionally low FIX activity and antigen levels [130]. In particular, secreted protein levels of the R294Q variant significantly increased (up to ~6 fold) upon treatment of stably-expressing cells with Na-PBA, a finding that was further confirmed at the intracellular level by the observation of improved trafficking to the Golgi compartment. Noticeably, a parallel increase in coagulant activity was also observed, with a dose-dependent shortening of coagulation times (up to ~18 s), which corresponded to an increase in coagulant activity levels from 0.5% (untreated) to ~3% upon treatment with Na-PBA. Taking into account the low therapeutic threshold, these levels, if cautiously translated to patients, would be sufficient to favor the transition from a severe/moderate to a moderate/mild phenotype, with potential alleviation of the bleeding tendency.

Overall, interventions at the translational and post-translational levels have the advantage that these approaches would modulate and/or rescue protein biosynthesis while maintaining the gene expression regulation in the physiological tissues only. However, major concerns limiting the therapeutic use of readthrough-inducing drugs such as gentamycin, or aminoglycosides in general, are the severe complications, such as kidney damage and hearing loss, related to their administration [208]. Interestingly, a delivery system based on encapsulated gentamycin reduced ototoxicity and increased dystrophin-positive fibers in mouse skeletal muscle cells in the mdx model of Duchenne muscular dystrophy [209]. Nevertheless, the development of aminoglycoside derivatives and non-aminoglycoside compounds was boosted by the need for less toxicity as well as improved selectivity and efficacy [210,211]. The unique example of a readthrough-inducing compound that has entered a clinical trial is PTC124 (Ataluren), orally administered in patients with hemophilia A and B, whose potential in treating bleeding disorders was precluded to be established due to suspension at phase 2a (Identifier NCT00947193) without data reports.

On the other hand, findings on chaperone-like compounds suggest that these molecules might represent an alternative approach to correct, with different degrees of responsiveness, a fraction of protein variants with defects in folding or intracellular processing. It is worth noting that the orally-administered NaPBA, albeit for different purposes [212], is approved for use in humans, which makes this compound a candidate as a potentially alternative therapeutic strategy.

Overall, these encouraging results for interventions at translational (ribosome readthrough) or post-translational (chaperone-like compounds) levels might be considered in light of the limits of non-standard molecules, which act only on responsive (nonsense or missense) mutations, and thus being mutation-specific, with benefits for diseases with a low therapeutic threshold.

## 6. Conclusions

Knowledge of the molecular bases of diseases such as coagulation factor disorders allowed the researchers to design new tailored therapeutic approaches at transcriptional, post-transcriptional (modulation of splicing), translational (induction of ribosome readthrough) or post-translational (folding correction) levels. These strategies are essentially based on specific gene features or molecular defects caused by splicing and nonsense/missense variants as well as nucleotide changes within the promoter sequence, which are collectively responsible for ~25% of severe coagulation factor deficiencies. Experimental evidences in both cellular and mouse models of disease showed the ability of these new strategies to produce a moderate to mild rescue of the expression levels of coagulation factors, which would result in a significant amelioration of the clinical phenotype if translated into patients. While a direct comparison of the correction efficacy of the different approaches is very difficult, the use of readthrough-inducing or of chaperone-like drugs appears to guarantee the lowest effect on protein expression that, for several disorders other than coagulopathies, might not achieve the therapeutic threshold. On the other hand, approaches at the RNA levels (splicing/AONs/siRNA), in the most favorable contexts, might completely rescue gene expression. An intermediate rescue extent has been obtained with genome editing techniques.

Collectively, the noticeable progresses in the design of tailored therapeutic strategies acting at multiple levels of gene expression (from DNA to RNA and protein), as well as in the production of viral vectors able to deliver them in a tissue-specific manner, generate an intriguing future in which further studies will be aimed at demonstrating their clinical translatability.

## Figures and Tables

**Table 1:** Coagulation factor deficiencies: genetics, severe symptoms and treatment options.

Pattern of Inheritance	Deficientfactor	OMIM* Number	Prevalence in the Population	Gene (Chromosome)	Severe Clinical Symptoms	Treatment Options**
X-linked	factor VIII^§^	306700	1:5,000	*F8* (Xq28)	Hemarthrosis, Intracranical hemorrhage	pdFVIII, rFVIII, EHL-FVIII
	factor IX^§^	306900	1:30,000	*F9* (Xq27.1)	Hemarthrosis, Intracranical hemorrhage	pdFIX, rFIX, EHL-FIX
Autosomal	Fibrinogen	202400	1:1,000,000	*FGA* (4q31.3)	Thrombosis, Umbilical stump bleeding, Mucocutaneous bleeding	FFP, pd Fibrinogen
				*FGB* (4q31.3)		
				*FGG* (4q32.1)		
	Prothrombin^†^	613679	1:2,000,000	*F2* (11p11.2)	Mucosal bleeding, Hemarthrosis, Intracranical hemorrhage	FFP, PCC
	factor V	227400	1:1,000,000	*F5* (1q24.2)	Epistaxis, Muscle hematoma	FFP
	factor VII^†§^	227500	1:500,000	*F7* (13q24)	Intracranical hemorrhage, Hemarthrosis	FFP, pdFVII, rFVIIa
	factor X^†^	227600	1:1,000,000	*F10* (13q34)	Gastrointesinal bleeding, Intracranical hemorrhage	FFP, PCC, pdFX/FIX, pdFX
	factor XI	612416	1:1,000,000	*F11* (4q35.2)	Post-trauma bleed	FFP, pdFXI
	factor XIII	613225	1:2,000,000	*F13A* (6q25.1)	Delayed wound healing, Intracranical hemorrhage, Miscarriages	FFP, pdFXIII, rFXIII-A
		613235		*F13B* (1q31.3)		

* OMIM, Online Mendelian Inheritance in Man (https://www.omim.org/); ** pd, plasma-derived; r, recombinant; EHL, enhanced half-life; FFP, fresh frozen plasma; PCC, prothrombin complex concentrate; † Factors whose complete deficiency is virtually incompatible with life; § Trials for gene therapy (excepting FVII deficiency, in which gene therapy has been characterized in animal models).

**Table 2:** Evaluation of drug-induced readthrough over nonsense mutations in FVIII, FIX and FVII deficiencies.

	Mutation (HGVS)*	Mutation (Legacy)**	Sense Codon	PTC Sequence Context	Predicted Amino Acid Insertion†	Cellular Model		Mouse Model		Patients		Ref
						Drug	Response	Drug	Response	Drug	Response	
*F8*	p.R446X	R427X	CGA	UGA-U	W / C / R	-	-	-	-	Gentamycin	No	[158]
	p.S1414X	S1395X	TCA	UAA-U	Y / Q / K	-	-	-	-	Gentamycin	1.6%	[158]
	p.R2135X	R2116X	CGA	UGA-G	W / C / R	-	-	-	-	Gentamycin	No	[158]
*F9*	p.G21X	G(-26)X	GGA	UGA-U	W / C / R	Geneticin	4%	-	-	-	-	[160]
	p.R75X	R29X	CGA	UGA-G	W / C / R	Geneticin	No	Geneticin / Gentamycin	No	-	-	[159,161]
	p.L103X	L57X	UUA	UAA-A	Y / Q / K	Geneticin	No	-	-	-	-	[161]
	p.R162X	R116X	CGA	UGA-C	W / C / R	Geneticin	~1%	-	-	-	-	[161]
	p.W240X	R194X	UGG	UGA-C	W / C / R	Geneticin	5–6%	-	-	-	-	[161]
	p.R294X	R248X	CGA	UGA-A	W / C / R	Geneticin	0.5–1%	-	-	-	-	[161]
	p.R298X	R252X	CGA	UGA-A	W / C / R	Geneticin	No	-	-	Gentamycin	No	[158,161]
	p.R379X	R333X	CGA	UGA-G	W / C / R	Geneticin	1%	-	-	Gentamycin	2% / No	[158,162]
	p.Q370X	R324X	CAG	UAG-U	Y / Q / K	Geneticin	~1%	-	-	-	-	[161]
	p.R384X	R338X	CGA	UGA-U	W / C / R	Geneticin	7-8%	Geneticin / Gentamycin	-	-	-	[159,161]
*F7*	p.S112X	S52X	UCA	UGA-A	W / C / R	Geneticin	2–3%	-	-	-	-	[163]
	p.C132X	C72X	UGC	UGA-C	W / C / R	Geneticin	12–13%	-	-	-	-	[163]
	p.K376X	K316X	AAG	UAG-G	Y / Q / K	Geneticin / Gentamycin	>3% / >2%	-	-	Gentamycin	sub-therapeutic	[164,165]
	p.W424X	W364X	UGG	UGA-G	W / C / R	Geneticin / Gentamycin	>3% / >2%	-	-	Gentamycin	sub-therapeutic	[164,165]

* Based on Human Genome Variation Society (HGVS) nomenclature numbering initiation methionine as +1 residue; ** Based on amino acid numbering of the secreted mature protein; † Predicted amino acid insertions as a function of the stop codon type.

## References

[1] Monroe D.M., Hoffman M. (2006). What Does It Take to Make the Perfect Clot?. Arterioscler. Thromb. Vasc. Biol..

[2] Bolton-Maggs P.H.B., Pasi K.J. (2003). Haemophilias A and B. Lancet.

[3] Giannelli F., Green P.M., Sommer S.S., Poon M.-C., Ludwig M., Schwaab R., Reitsma P.H., Goossens M., Yoshioka A., Figueiredo M.S. (1997). Haemophilia B: Database of point mutations and short additions and deletions, 7th edition. Nucleic Acids Res..

[4] Payne A.B., Miller C.H., Kelly F.M., Michael Soucie J., Craig Hooper W. (2012). The CDC Hemophilia A Mutation Project (CHAMP) Mutation List: A New Online Resource. Hum. Mutat..

[5] Li T., Miller C.H., Payne A.B., Craig Hooper W. (2013). The CDC Hemophilia B mutation project mutation list: A new online resource. Mol. Genet. Genomic Med..

[6] Antonarakis S.E., Kazazian H.H., Tuddenham E.G.D. (1995). Molecular etiology of factor VIII deficiency in hemophilia A. Hum. Mutat..

[7] Mannucci P.M. (2004). Recessively inherited coagulation disorders. Blood.

[8] Palla R., Peyvandi F., Shapiro A.D. (2015). Rare bleeding disorders: Diagnosis and treatment. Blood.

[9] Rosen E.D., Chan J.C.Y.Y., Idusogie E., Clotman F., Vlasuk G., Luther T., Jalbert L.R., Albrecht S., Zhong L., Lissens A. (1997). Mice lacking factor VII develop normally but suffer fatal perinatal bleeding. Nature.

[10] Dewerchin M., Liang Z., Moons L., Carmeliet P., Castellino F., Collen D., Rosen E. (2000). Blood Coagulation Factor X Deficiency Causes Partial Embryonic Lethality and Fatal Neonatal Bleeding in Mice. Thromb. Haemost..

[11] Xue J., Wu Q., Westfield L.A., Tuley E.A., Lu D., Zhang Q., Shim K., Zheng X., Sadler J.E. (1998). Incomplete embryonic lethality and fatal neonatal hemorrhage caused by prothrombin deficiency in mice. Proc. Natl. Acad. Sci. USA.

[12] Mullins E.S., Kombrinck K.W., Talmage K.E., Shaw M.A., Witte D.P., Ullman J.M., Degen S.J., Sun W., Flick M.J., Degen J.L. (2008). Genetic elimination of prothrombin in adult mice is not compatible with survival and results in spontaneous hemorrhagic events in both heart and brain. Blood.

[13] Salomon O., Steinberg D.M., Seligshon U. (2006). Variable bleeding manifestations characterize different types of surgery in patients with severe factor XI deficiency enabling parsimonious use of replacement therapy. Haemophilia.

[14] Menegatti M., Peyvandi F. (2018). Treatment of rare factor deficiencies other than hemophilia. Blood.

[15] Magee G., Zbrozek A. (2013). Fluid overload is associated with increases in length of stay and hospital costs: Pooled analysis of data from more than 600 US hospitals. Clin. Outcomes Res..

[16] Sorensen B., Spahn D.R., Innerhofer P., Spannagl M., Rossaint R. (2011). Clinical review: Prothrombin complex concentrates - evaluation of safety and thrombogenicity. Crit. Care.

[17] Shapiro A. (2016). Plasma-derived human factor X concentrate for on-demand and perioperative treatment in factor X-deficient patients: Pharmacology, pharmacokinetics, efficacy, and safety. Expert Opin. Drug Metab. Toxicol..

[18] Lovejoy A.E. (2006). Safety and pharmacokinetics of recombinant factor XIII-A2 administration in patients with congenital factor XIII deficiency. Blood.

[19] Kerlin B., Brand B., Inbal A., Halimeh S., Nugent D., Lundblad M., Tehranchi R. (2014). Pharmacokinetics of recombinant factor XIII at steady state in patients with congenital factor XIII A-subunit deficiency. J. Thromb. Haemost..

[20] Bulato C., Novembrino C., Anzoletti M.B., Spiezia L., Gavasso S., Berbenni C., Tagariello G., Farina C., Nardini I., Campello E. (2018). “In vitro” correction of the severe factor V deficiency-related coagulopathy by a novel plasma-derived factor V concentrate. Haemophilia.

[21] DiMichele D. (2007). Inhibitor development in haemophilia B: An orphan disease in need of attention. Br. J. Haematol..

[22] Franchini M., Mannucci P.M. (2011). Inhibitors of propagation of coagulation (factors VIII, IX and XI): A review of current therapeutic practice. Br. J. Clin. Pharmacol..

[23] Persson E., Bolt G., Steenstrup T.D., Ezban M. (2010). Recombinant coagulation factor VIIa – from molecular to clinical aspects of a versatile haemostatic agent. Thromb. Res..

[24] Kempton C.L., Meeks S.L. (2014). Toward optimal therapy for inhibitors in hemophilia. Blood.

[25] Mannucci P.M. (2015). Half-life extension technologies for haemostatic agents. Thromb. Haemost..

[26] Peyvandi F., Garagiola I., Biguzzi E. (2016). Advances in the treatment of bleeding disorders. J. Thromb. Haemost..

[27] Samelson-Jones B.J., Arruda V.R. (2019). Protein-Engineered Coagulation Factors for Hemophilia Gene Therapy. Mol. Ther. Methods Clin. Dev..

[28] Marcos-Contreras O.A., Smith S.M., Bellinger D.A., Raymer R.A., Merricks E., Faella A., Pavani G., Zhou S., Nichols T.C., High K.A. (2015). Sustained correction of FVII deficiency in dogs using AAV-mediated expression of zymogen FVII. Blood.

[29] Binny C., McIntosh J., Della Peruta M., Kymalainen H., Tuddenham E.G.D., Buckley S.M.K., Waddington S.N., McVey J.H., Spence Y., Morton C.L. (2012). AAV-mediated gene transfer in the perinatal period results in expression of FVII at levels that protect against fatal spontaneous hemorrhage. Blood.

[30] Carcao M.D., Aledort L. (2004). Prophylactic factor replacement in hemophilia. Blood Rev..

[31] Kontermann R.E. (2016). Half-life extended biotherapeutics. Expert Opin. Biol. Ther..

[32] Weimer T., Wormsbächer W., Kronthaler U., Lang W., Liebing U., Schulte S. (2008). Prolonged in-vivo half-life of factor VIIa by fusion to albumin. Thromb. Haemost..

[33] Weimer T., Kronthaler U., Lang W., Schulte S., Metzner H.J. (2009). Genetic fusion to albumin improves the pharmacokinetic properties of factor IX. Thromb. Haemost..

[34] Ferrarese M., Pignani S., Lombardi S., Balestra D., Bernardi F., Pinotti M., Branchini A. (2019). The carboxyl-terminal region of human coagulation factor X as a natural linker for fusion strategies. Thromb. Res..

[35] Millar D., Kemball-Cook G., McVey J., Tuddenham E., Mumford A., Attock G., Reverter J., Lanir N., Parapia L., Reynaud J. (2000). Molecular analysis of the genotype-phenotype relationship in factor VII deficiency. Hum. Genet..

[36] Millar D.S., Elliston L., Deex P., Krawczak M., Wacey A.I., Reynaud J., Nieuwenhuis H.K., Bolton-Maggs P., Mannucci P.M., Reverter J.C. (2000). Molecular analysis of the genotype-phenotype relationship in factor X deficiency. Hum. Genet..

[37] Shee C.D. (2005). Oxford Textbook of Medicine. J. R. Soc. Med..

[38] Chuah M.K., Evens H., VandenDriessche T. (2013). Gene therapy for hemophilia. J. Thromb. Haemost..

[39] High K.A. (2001). Gene Transfer as an Approach to Treating Hemophilia. Circ. Res..

[40] Pierce G.F., Lillicrap D., Pipe S.W., Vandendriessche T. (2007). Gene therapy, bioengineered clotting factors and novel technologies for hemophilia treatment. J. Thromb. Haemost..

[41] Nathwani A.C., Reiss U.M., Tuddenham E.G.D.D., Rosales C., Chowdary P., McIntosh J., Della Peruta M., Lheriteau E., Patel N., Raj D. (2014). Long-Term Safety and Efficacy of Factor IX Gene Therapy in Hemophilia B. N. Engl. J. Med..

[42] Mingozzi F., Büning H. (2015). Adeno-Associated Viral Vectors at the Frontier between Tolerance and Immunity. Front. Immunol..

[43] Mariani G., Herrmann F.H., Schulman S., Batorova A., Wulff K., Etro D., Dolce A., Auerswald G., Astermark J., Schved J.-F. (2003). Thrombosis in inherited factor VII deficiency. J. Thromb. Haemost..

[44] Bonetta L. (2009). RNA-Based Therapeutics: Ready for Delivery?. Cell.

[45] Du L., Gatti R.A. (2009). Progress toward therapy with antisense-mediated splicing modulation. Curr. Opin. Mol. Ther..

[46] Nlend Nlend R., Meyer K., Schümperli D. (2010). Repair of pre-mRNA splicing: Prospects for a therapy for spinal muscular atrophy. RNA Biol..

[47] Ward A.J., Cooper T.A. (2010). The pathobiology of splicing. J. Pathol..

[48] Hammond S.M., Wood M.J.A. (2011). Genetic therapies for RNA mis-splicing diseases. Trends Genet..

[49] Casari C., Pinotti M., Lancellotti S., Adinolfi E., Casonato A., De Cristofaro R., Bernardi F. (2010). The dominant-negative von Willebrand factor gene deletion p.P1127_C1948delinsR: Molecular mechanism and modulation. Blood.

[50] Gaj T., Gersbach C.A., Barbas C.F. (2013). ZFN, TALEN, and CRISPR/Cas-based methods for genome engineering. Trends Biotechnol..

[51] Sürün D., Kurrle N., Serve H., von Melchner H., Schnütgen F. (2017). High efficiency gene correction in hematopoietic cells by donor-template-free CRISPR/Cas9 genome editing. Exp. Hematol..

[52] Sadelain M., Papapetrou E.P., Bushman F.D. (2011). Safe harbours for the integration of new DNA in the human genome. Nat. Rev. Cancer.

[53] Li H., Haurigot V., Doyon Y., Li T., Wong S.Y., Bhagwat A.S., Malani N., Anguela X.M., Sharma R., Ivanciu L. (2011). In vivo genome editing restores haemostasis in a mouse model of haemophilia. Nature.

[54] Anguela X.M., Sharma R., Doyon Y., Miller J.C., Li H., Haurigot V., Rohde M.E., Wong S.Y., Davidson R.J., Zhou S. (2013). Robust ZFN-mediated genome editing in adult hemophilic mice. Blood.

[55] Huai C., Jia C., Sun R., Xu P., Min T., Wang Q., Zheng C., Chen H., Lu D. (2017). CRISPR/Cas9-mediated somatic and germline gene correction to restore hemostasis in hemophilia B mice. Hum. Genet..

[56] Lyu C., Shen J., Wang R., Gu H., Zhang J., Xue F., Liu X., Liu W., Fu R., Zhang L.L.L.L. (2018). Targeted genome engineering in human induced pluripotent stem cells from patients with hemophilia B using the CRISPR-Cas9 system. Stem Cell Res. Ther..

[57] Liu X., Wang Y., Hu Z., Wu L., Zhao J., Feng M., Li Z., Liang D., Zhang Y., Zhou M. (2018). Paired CRISPR/Cas9 Nickases Mediate Efficient Site-Specific Integration of F9 into rDNA Locus of Mouse ESCs. Int. J. Mol. Sci..

[58] Sharma R., Anguela X.M., Doyon Y., Wechsler T., DeKelver R.C., Sproul S., Paschon D.E., Miller J.C., Davidson R.J., Shivak D. (2015). In vivo genome editing of the albumin locus as a platform for protein replacement therapy. Blood.

[59] Barzel A., Paulk N.K., Shi Y., Huang Y., Chu K., Zhang F., Valdmanis P.N., Spector L.P., Porteus M.H., Gaensler K.M. (2014). Promoterless gene targeting without nucleases ameliorates haemophilia B in mice. Nature.

[60] Ohmori T., Nagao Y., Mizukami H., Sakata A., Muramatsu S.I., Ozawa K., Tominaga S.I., Hanazono Y., Nishimura S., Nureki O. (2017). CRISPR/Cas9-mediated genome editing via postnatal administration of AAV vector cures haemophilia B mice. Sci. Rep..

[61] Park C.-Y., Kim J., Kweon J., Son J.S., Lee J.S., Yoo J.-E., Cho S.-R., Kim J.-H., Kim J.-S., Kim D.-W. (2014). Targeted inversion and reversion of the blood coagulation factor 8 gene in human iPS cells using TALENs. Proc. Natl. Acad. Sci. USA.

[62] Park C.Y., Kim D.H., Son J.S., Sung J.J., Lee J., Bae S., Kim J.H., Kim D.W., Kim J.S. (2015). Functional Correction of Large Factor VIII Gene Chromosomal Inversions in Hemophilia A Patient-Derived iPSCs Using CRISPR-Cas9. Cell Stem Cell.

[63] Guan Y., Ma Y., Li Q., Sun Z., Ma L., Wu L., Wang L., Zeng L., Shao Y., Chen Y. (2016). CRISPR/Cas9-mediated somatic correction of a novel coagulator factor IX gene mutation ameliorates hemophilia in mouse. EMBO Mol. Med..

[64] Barbon E., Pignani S., Branchini A., Bernardi F., Pinotti M., Bovolenta M. (2016). An engineered tale-transcription factor rescues transcription of factor VII impaired by promoter mutations and enhances its endogenous expression in hepatocytes. Sci. Rep..

[65] Arbini A.A., Pollak E.S., Bayleran J.K., High K.A., Bauer K.A. (1997). Severe factor VII deficiency due to a mutation disrupting a hepatocyte nuclear factor 4 binding site in the factor VII promoter. Blood.

[66] Carew J.A., Pollak E.S., High K.A., Bauer K.A. (1998). Severe Factor VII Deficiency Due to a Mutation Disrupting an Sp1 Binding Site in the Factor VII Promoter. Blood.

[67] Pignani S., Zappaterra F., Barbon E., Follenzi A., Bovolenta M., Bernardi F., Branchini A., Pinotti M. (2019). Tailoring the CRISPR system to transactivate coagulation gene promoters in normal and mutated contexts. Biochim. Biophys. Acta Gene Regul. Mech..

[68] Lander E.S., Linton L.M., Birren B., Nusbaum C., Zody M.C., Baldwin J., Devon K., Dewar K., Doyle M., Fitzhugh W. (2001). Initial sequencing and analysis of the human genome. Nature.

[69] Ramanouskaya T.V., Grinev V.V. (2017). The determinants of alternative RNA splicing in human cells. Mol. Genet. Genom..

[70] Pan Q., Shai O., Lee L.J., Frey B.J., Blencowe B.J. (2008). Deep surveying of alternative splicing complexity in the human transcriptome by high-throughput sequencing. Nat. Genet..

[71] Barbosa-Morais N.L., Irimia M., Pan Q., Xiong H.Y., Gueroussov S., Lee L.J., Slobodeniuc V., Kutter C., Watt S., Colak R. (2012). The Evolutionary Landscape of Alternative Splicing in Vertebrate Species. Science (80-).

[72] Merkin J., Russell C., Chen P., Burge C.B. (2012). Evolutionary Dynamics of Gene and Isoform Regulation in Mammalian Tissues. Science (80-).

[73] Bentley D.L. (2014). Coupling mRNA processing with transcription in time and space. Nat. Rev. Genet..

[74] Naftelberg S., Schor I.E., Ast G., Kornblihtt A.R. (2015). Regulation of Alternative Splicing Through Coupling with Transcription and Chromatin Structure. Annu. Rev. Biochem..

[75] Lee Y., Rio D.C. (2015). Mechanisms and Regulation of Alternative Pre-mRNA Splicing. Annu. Rev. Biochem..

[76] Sumanasekera C., Watt D.S., Stamm S. (2008). Substances that can change alternative splice-site selection. Biochem. Soc. Trans..

[77] Montes M., Becerra S., Sánchez-Álvarez M., Suñé C. (2012). Functional coupling of transcription and splicing. Gene.

[78] Gómez Acuña L.I., Fiszbein A., Alló M., Schor I.E., Kornblihtt A.R. (2013). Connections between chromatin signatures and splicing. Wiley Interdiscip. Rev. RNA.

[79] Kornblihtt A.R., De La Mata M., Fededa J.P., Muñoz M.J., Nogués G. (2004). Multiple links between transcription and splicing. RNA.

[80] Shukla S., Oberdoerffer S. (2012). Co-transcriptional regulation of alternative pre-mRNA splicing. Biochim. Biophys. Acta Gene Regul. Mech..

[81] Krawczak M., Thomas N.S.T., Hundrieser B., Mort M., Wittig M., Hampe J., Cooper D.N. (2007). Single base-pair substitutions in exon-intron junctions of human genes: Nature, distribution, and consequences for mRNA splicing. Hum. Mutat..

[82] Baralle D., Buratti E. (2017). RNA splicing in human disease and in the clinic. Clin. Sci..

[83] Tazi J., Bakkour N., Stamm S. (2009). Alternative splicing and disease. Biochim. Biophys. Acta Mol. Basis Dis..

[84] Faustino N.A., Cooper T.A. (2003). Pre-mRNA splicing and human disease. Genes Dev..

[85] Rogalska M.E., Tajnik M., Licastro D., Bussani E., Camparini L., Mattioli C., Pagani F. (2016). Therapeutic activity of modified U1 core spliceosomal particles. Nat. Commun..

[86] Balestra D., Barbon E., Scalet D., Cavallari N., Perrone D., Zanibellato S., Bernardi F., Pinotti M. (2015). Regulation of a strong F9 cryptic 5′ss by intrinsic elements and by combination of tailored U1snRNAs with antisense oligonucleotides. Hum. Mol. Genet..

[87] Donadon I., McVey J.H., Garagiola I., Branchini A., Mortarino M., Peyvandi F., Bernardi F., Pinotti M. (2017). Clustered F8 missense mutations cause hemophilia A by combined alteration of splicing and protein biosynthesis and activity. Haematologica.

[88] Cavallari N., Balestra D., Branchini A., Maestri I., Chuamsunrit A., Sasanakul W., Mariani G., Pagani F., Bernardi F., Pinotti M. (2012). Activation of a cryptic splice site in a potentially lethal coagulation defect accounts for a functional protein variant. Biochim. Biophys. Acta Mol. Basis Dis..

[89] Grodecká L., Buratti E., Freiberger T. (2017). Mutations of pre-mRNA splicing regulatory elements: Are predictions moving forward to clinical diagnostics?. Int. J. Mol. Sci..

[90] Lenting P.J., van Mourik J.A., Mertens K. (1998). Determinants of the inherent strength of human 5′ splice sites. Blood.

[91] Tan J., Ho J.X.J.X.J., Zhong Z., Luo S., Chen G., Roca X. (2016). Noncanonical registers and base pairs in human 5′ splice-site selection. Nucleic Acids Res..

[92] Roca X., Akerman M., Gaus H., Berdeja A., Bennett C.F., Krainer A.R. (2012). Widespread recognition of 5’ splice sites by noncanonical base-pairing to U1 snRNA involving bulged nucleotides. Genes Dev..

[93] Krawczak M., Reiss J., Cooper D.N. (1992). The mutational spectrum of single base-pair substitutions in mRNA splice junctions of human genes: Causes and consequences. Hum. Genet..

[94] Zhuang Y., Weiner A.M. (1986). A compensatory base change in U1 snRNA suppresses a 5′ splice site mutation. Cell.

[95] Baralle M. (2003). Identification of a mutation that perturbs NF1 agene splicing using genomic DNA samples and a minigene assay. J. Med. Genet..

[96] Susani L., Pangrazio A., Sobacchi C., Taranta A., Mortier G., Savarirayan R., Villa A., Orchard P., Vezzoni P., Albertini A. (2004). TCIRG1-dependent recessive osteopetrosis: Mutation analysis, functional identification of the splicing defects, andin vitro rescue by U1 snRNA. Hum. Mutat..

[97] Pinotti M., Balestra D., Rizzotto L., Maestri I., Pagani F., Bernardi F. (2009). Rescue of coagulation factor VII function by the U1+5A snRNA. Blood.

[98] Pinotti M., Rizzotto L., Balestra D., Lewandowska M.A., Cavallari N., Marchetti G., Bernardi F., Pagani F. (2008). U1-snRNA-mediated rescue of mRNA processing in severe factor VII deficiency. Blood.

[99] Tanner G., Glaus E., Barthelmes D., Ader M., Fleischhauer J., Pagani F., Berger W., Neidhardt J. (2009). Therapeutic strategy to rescue mutation-induced exon skipping in rhodopsin by adaptation of U1 snRNA. Hum. Mutat..

[100] Hartmann L., Neveling K., Borkens S., Schneider H., Freund M., Grassman E., Theiss S., Wawer A., Burdach S., Auerbach A.D. (2010). Correct mRNA Processing at a Mutant TT Splice Donor in FANCC Ameliorates the Clinical Phenotype in Patients and Is Enhanced by Delivery of Suppressor U1 snRNAs. Am. J. Hum. Genet..

[101] Schmid F., Glaus E., Barthelmes D., Fliegauf M., Gaspar H., Nürnberg G., Nürnberg P., Omran H., Berger W., Neidhardt J. (2011). U1 snRNA-mediated gene therapeutic correction of splice defects caused by an exceptionally mild BBS mutation. Hum. Mutat..

[102] Glaus E., Schmid F., Da Costa R., Berger W., Neidhardt J. (2011). Gene Therapeutic Approach Using Mutation-adapted U1 snRNA to Correct a RPGR Splice Defect in Patient-derived Cells. Mol. Ther..

[103] Borensztajn K., Sobrier M.-L.L., Duquesnoy P., Fischer A.-M.M., Tapon-Bretaudière J., Amselem S. (2006). Oriented Scanning Is the Leading Mechanism Underlying 5′ Splice Site Selection in Mammals. PLoS Genet..

[104] Balestra D., Faella A., Margaritis P., Cavallari N., Pagani F., Bernardi F., Arruda V.R., Pinotti M. (2014). An engineered U1 small nuclear RNA rescues splicing-defective coagulation F7 gene expression in mice. J. Thromb. Haemost..

[105] Tajnik M., Rogalska M.E., Bussani E., Barbon E., Balestra D., Pinotti M., Pagani F. (2016). Molecular Basis and Therapeutic Strategies to Rescue Factor IX Variants That Affect Splicing and Protein Function. PLoS Genet..

[106] Dal Mas A., Fortugno P., Donadon I., Levati L., Castiglia D., Pagani F. (2015). Exon-Specific U1s Correct SPINK5Exon 11 Skipping Caused by a Synonymous Substitution that Affects a Bifunctional Splicing Regulatory Element. Hum. Mutat..

[107] Dal Mas A., Rogalska M.E.E., Bussani E., Pagani F., Dal Mas A., Rogalska M.E.E., Bussani E., Pagani F. (2015). Improvement of SMN2 Pre-mRNA Processing Mediated by Exon-Specific U1 Small Nuclear RNA. Am. J. Hum. Genet..

[108] Nizzardo M., Simone C., Dametti S., Salani S., Ulzi G., Pagliarani S., Rizzo F., Frattini E., Pagani F., Bresolin N. (2015). Spinal muscular atrophy phenotype is ameliorated in human motor neurons by SMN increase via different novel RNA therapeutic approaches. Sci. Rep..

[109] van der Woerd W.L., Mulder J., Pagani F., Beuers U., Houwen R.H.J., van de Graaf S.F.J. (2015). Analysis of aberrant pre-messenger RNA splicing resulting from mutations in ATP8B1 and efficient in vitro rescue by adapted U1 small nuclear RNA. Hepatology.

[110] Donadon I., Pinotti M., Rajkowska K., Pianigiani G., Barbon E., Morini E., Motaln H., Rogelj B., Mingozzi F., Slaugenhaupt S.A. (2018). Exon-specific U1 snRNAs improve ELP1 exon 20 definition and rescue ELP1 protein expression in a familial dysautonomia mouse model. Hum. Mol. Genet..

[111] Scalet D., Balestra D., Rohban S., Bovolenta M., Perrone D., Bernardi F., Campaner S., Pinotti M. (2017). Exploring Splicing-Switching Molecules For Seckel Syndrome Therapy. Biochim. Biophys. Acta - Mol. Basis Dis..

[112] Fernandez Alanis E., Pinotti M., Dal Mas A., Balestra D., Cavallari N., Rogalska M.E., Bernardi F., Pagani F., Alanis E.F., Pinotti M. (2012). An exon-specific U1 small nuclear RNA (snRNA) strategy to correct splicing defects. Hum. Mol. Genet..

[113] Balestra D., Scalet D., Pagani F., Rogalska M.E., Mari R., Bernardi F., Pinotti M. (2016). An Exon-Specific U1snRNA Induces a Robust Factor IX Activity in Mice Expressing Multiple Human FIX Splicing Mutants. Mol. Ther. Nucleic Acids.

[114] Martínez-Pizarro A., Dembic M., Pérez B., Andresen B.S., Desviat L.R. (2018). Intronic PAH gene mutations cause a splicing defect by a novel mechanism involving U1snRNP binding downstream of the 5’ splice site. PLoS Genet..

[115] Scalet D., Sacchetto C., Bernardi F., Pinotti M., Van De Graaf S.F.J., Balestra D. (2018). The somatic FAH C.1061C>A change counteracts the frequent FAH c.1062+5G>A mutation and permits U1snRNA-based splicing correction. J. Hum. Genet..

[116] Scalet D., Maestri I., Branchini A., Bernardi F., Pinotti M., Balestra D. (2019). Disease-causing variants of the conserved +2T of 5′ splice sites can be rescued by engineered U1snRNAs. Hum. Mutat..

[117] Sheth N., Roca X., Hastings M.L., Roeder T., Krainer A.R., Sachidanandam R. (2006). Comprehensive splice-site analysis using comparative genomics. Nucleic Acids Res..

[118] Thanaraj T.A. (2001). Human GC-AG alternative intron isoforms with weak donor sites show enhanced consensus at acceptor exon positions. Nucleic Acids Res..

[119] Nuzzo F., Bulato C., Nielsen B.I., Lee K., Wielders S.J., Simioni P., Key N.S., Castoldi E. (2014). Characterization of an apparently synonymous F5 mutation causing aberrant splicing and factor V deficiency. Haemophilia.

[120] Nuzzo F., Radu C., Baralle M., Spiezia L., Hackeng T.M., Simioni P., Castoldi E. (2013). Antisense-based RNA therapy of factor V deficiency: In vitro and ex vivo rescue of a F5 deep-intronic splicing mutation. Blood.

[121] Davis R.L., Homer V.M., George P.M., Brennan S.O. (2009). A deep intronic mutation in FGB creates a consensus exonic splicing enhancer motif that results in afibrinogenemia caused by aberrant mRNA splicing, which can be corrected in vitro with antisense oligonucleotide treatment. Hum. Mutat..

[122] de Jong A., Dirven R.J., Oud J.A., Tio D., van Vlijmen B.J.M., Eikenboom J. (2018). Correction of a dominant-negative von Willebrand factor multimerization defect by small interfering RNA-mediated allele-specific inhibition of mutant von Willebrand factor. J. Thromb. Haemost..

[123] Sehgal A., Barros S., Ivanciu L., Cooley B., Qin J., Racie T., Hettinger J., Carioto M., Jiang Y., Brodsky J. (2015). An RNAi therapeutic targeting antithrombin to rebalance the coagulation system and promote hemostasis in hemophilia. Nat. Med..

[124] Pasi K.J., Rangarajan S., Georgiev P., Mant T., Creagh M.D., Lissitchkov T., Bevan D., Austin S., Hay C.R., Hegemann I. (2017). Targeting of Antithrombin in Hemophilia A or B with RNAi Therapy. N. Engl. J. Med..

[125] Rodnina M.V., Wintermeyer W. (2001). Ribosome fidelity: tRNA discrimination, proofreading and induced fit. Trends Biochem. Sci..

[126] Cochella L., Green R. (2005). Fidelity in protein synthesis. Curr. Biol..

[127] Bereczky Z., Bardos H., Komaromi I., Kiss C., Haramura G., Ajzner E., Adany R., Muszbek L. (2008). Factor XDebrecen: Gly204Arg mutation in factor X causes the synthesis of a non-secretable protein and severe factor X deficiency. Haematologica.

[128] Branchini A., Campioni M., Mazzucconi M.G., Biondo F., Mari R., Bicocchi M.P., Bernardi F., Pinotti M. (2013). Replacement of the Y450 (c234) phenyl ring in the carboxyl-terminal region of coagulation factor IX causes pleiotropic effects on secretion and enzyme activity. FEBS Lett..

[129] Baroni M., Pavani G., Pinotti M., Branchini A., Bernardi F., Camire R.M. (2015). Asymmetric processing of mutant factor X Arg386Cys reveals differences between intrinsic and extrinsic pathway activation. Biochim. Biophys. Acta Proteins Proteomics.

[130] Pignani S., Todaro A., Ferrarese M., Marchi S., Lombardi S., Balestra D., Pinton P., Bernardi F., Pinotti M., Branchini A. (2018). The chaperone-like sodium phenylbutyrate improves factor IX intracellular trafficking and activity impaired by the frequent p.R294Q mutation. J. Thromb. Haemost..

[131] Kisselev L.L., Buckingham R.H. (2000). Translational termination comes of age. Trends Biochem. Sci..

[132] Mort M., Ivanov D., Cooper D.N., Chuzhanova N.A. (2008). A meta-analysis of nonsense mutations causing human genetic disease. Hum. Mutat..

[133] Nagy E., Maquat L.E. (1998). A rule for termination-codon position within intron-containing genes: When nonsense affects RNA abundance. Trends Biochem. Sci..

[134] Khajavi M., Inoue K., Lupski J.R. (2006). Nonsense-mediated mRNA decay modulates clinical outcome of genetic disease. Eur. J. Hum. Genet..

[135] Rospert S., Rakwalska M., Dubaquié Y. (2005). Polypeptide chain termination and stop codon readthrough on eukaryotic ribosomes. Rev. Physiol. Biochem. Pharmacol..

[136] Roy B., Leszyk J.D., Mangus D.A., Jacobson A. (2015). Nonsense suppression by near-cognate tRNAs employs alternative base pairing at codon positions 1 and 3. Proc. Natl. Acad. Sci. USA.

[137] Brown C.M., Stockwell P.A., Trotman C.N.A., Tate W.P. (1990). Sequence analysis suggests that tetra-nucleotides signal the termination of protein synthesis in eukaryotes. Nucleic Acids Res..

[138] Manuvakhova M., Keeling K., Bedwell D.M. (2000). Aminoglycoside antibiotics mediate context-dependent suppression of termination codons in a mammalian translation system. RNA.

[139] Cobucci-Ponzano B., Rossi M., Moracci M. (2004). Recoding in Archaea. Mol. Microbiol..

[140] Beier H. (2001). Misreading of termination codons in eukaryotes by natural nonsense suppressor tRNAs. Nucleic Acids Res..

[141] Böck A., Forchhammer K., Heider J., Leinfelder W., Sawers G., Veprek B., Zinoni F. (1991). Selenocysteine: The 21st amino acid. Mol. Microbiol..

[142] Berry M.J., Small-Howard A.L. (2005). Unique features of selenocysteine incorporation function within the context of general eukaryotic translational processes. Biochem. Soc. Trans..

[143] James C.M., Ferguson T.K., Leykam J.F., Krzycki J.A. (2001). The Amber Codon in the Gene Encoding the Monomethylamine Methyltransferase Isolated fromMethanosarcina barkeriIs Translated as a Sense Codon. J. Biol. Chem..

[144] Atkins J.F., Gesteland R. (2002). Biochemistry: The 22nd amino acid. Science (80-).

[145] Burke J.F., Mogg A.E. (1985). Suppression of a nonsense mutation in mammalian cellsin vivoby the aminoglycoside anthiotics G–418 and paromomycin. Nucleic Acids Res..

[146] Ogle J.M., Brodersen D.E., Clemons W.M. J., Tarry M.J., Carter A.P., Ramakrishnan V. (2001). Recognition of cognate transfer RNA by the 30S ribosomal subunit. Science (80-).

[147] Howard M., Frizzell R.A., Bedwell D.M. (1996). Aminoglycoside antibiotics restore CFTR function by overcoming premature stop mutations. Nat. Med..

[148] Keeling K.M., Xue X., Gunn G., Bedwell D.M. (2014). Therapeutics Based on Stop Codon Readthrough. Annu. Rev. Genomics Hum. Genet..

[149] Keeling K. (2016). Nonsense Suppression as an Approach to Treat Lysosomal Storage Diseases. Diseases.

[150] Blanchet S., Cornu D., Argentini M., Namy O. (2014). New insights into the incorporation of natural suppressor tRNAs at stop codons in Saccharomyces cerevisiae. Nucleic Acids Res..

[151] Roy B., Friesen W.J., Tomizawa Y., Leszyk J.D., Zhuo J., Johnson B., Dakka J., Trotta C.R., Xue X., Mutyam V. (2016). Ataluren stimulates ribosomal selection of near-cognate tRNAs to promote nonsense suppression. Proc. Natl. Acad. Sci. USA.

[152] Branchini A., Rizzotto L., Mariani G., Napolitano M., Lapecorella M., Giansily-Blaizot M., Mari R., Canella A., Pinotti M., Bernardi F. (2012). Natural and engineered carboxy-terminal variants: Decreased secretion and gain-of-function result in asymptomatic coagulation factor VII deficiency. Haematologica.

[153] Branchini A., Baroni M., Pfeiffer C., Batorova A., Giansily-Blaizot M., Schved J.F., Mariani G., Bernardi F., Pinotti M. (2014). Coagulation factor VII variants resistant to inhibitory antibodies. Thromb. Haemost..

[154] Borhany M., Buthiau D., Rousseau F., Guillot O., Naveena F., Abid M., Shamsi T., Giansily-Blaizot M. (2018). Genotyping of five Pakistani patients with severe inherited factor X deficiency. Blood Coagul. Fibrinolysis.

[155] Ferrarese M., Baroni M., Della Valle P., Spiga I., Poloniato A., D’Angelo A., Pinotti M., Bernardi F., Branchini A. (2019). Missense changes in the catalytic domain of coagulation factor X account for minimal function preventing a perinatal lethal condition. Haemophilia.

[156] Branchini A., Baroni M., Burini F., Puzzo F., Nicolosi F., Mari R., Gemmati D., Bernardi F., Pinotti M. (2015). The carboxyl-terminal region is NOT essential for secreted and functional levels of coagulation factor X. J. Thromb. Haemost..

[157] Pinotti M., Caruso P., Canella A., Campioni M., Tagariello G., Castaman G., Giacomelli S., Belvini D., Bernardi F. (2012). Ribosome readthrough accounts for secreted full-length factor IX in hemophilia B patients with nonsense mutations. Hum. Mutat..

[158] James P.D. (2005). Aminoglycoside suppression of nonsense mutations in severe hemophilia. Blood.

[159] Yang C., Feng J., Song W., Wang J., Tsai B., Zhang Y., Scaringe W.A., Hill K.A., Margaritis P., High K.A. (2007). A mouse model for nonsense mutation bypass therapy shows a dramatic multiday response to geneticin. Proc. Natl. Acad. Sci. USA.

[160] Ferrarese M., Testa M.F., Balestra D., Bernardi F., Pinotti M., Branchini A. (2018). Secretion of wild-type factor IX upon readthrough over F9 pre-peptide nonsense mutations causing hemophilia B. Hum. Mutat..

[161] Branchini A., Ferrarese M., Campioni M., Castaman G., Mari R., Bernardi F., Pinotti M. (2017). Specific factor IX mRNA and protein features favor drug-induced readthrough over recurrent nonsense mutations. Blood.

[162] Simioni P., Tormene D., Tognin G., Gavasso S., Bulato C., Iacobelli N.P., Finn J.D., Spiezia L., Radu C., Arruda V.R. (2009). X-Linked Thrombophilia with a Mutant Factor IX (Factor IX Padua). N. Engl. J. Med..

[163] Branchini A., Ferrarese M., Lombardi S., Mari R., Bernardi F., Pinotti M. (2016). Differential functional readthrough over homozygous nonsense mutations contributes to the bleeding phenotype in coagulation factor VII deficiency. J. Thromb. Haemost..

[164] Pinotti M., Rizzotto L., Pinton P., Ferraresi P., Chuansumrit A., Charoenkwan P., Marchetti G., Rizzuto R., Mariani G., Bernardi F. (2006). Intracellular readthrough of nonsense mutations by aminoglycosides in coagulation factor VII. J. Thromb. Haemost..

[165] Pinotti M., Rizzotto L., Chuansumrit A., Mariani G., Bernardi F. (2006). Gentamicin induces sub-therapeutic levels of coagulation factor VII in patients with nonsense mutations. J. Thromb. Haemost..

[166] Giansily-Blaizot M., Aguilar-Martinez P., Briquel M.-E., d’Oiron R., De Maistre E., Epelbaum S., Schved J.-F. (2003). Two novel cases of cerebral haemorrhages at the neonatal period associated with inherited factor VII deficiency, one of them revealing a new nonsense mutation (Ser52Stop). Blood Coagul. Fibrinolysis.

[167] Chafa O., Fischer A.M., Reghis A., Tapon-Bretaudiere J. (2005). Homozygous nonsense mutation (Cys72→stop) in the human F7 gene: A not life-threatening mutation despite the absence of circulating factor VII. J. Thromb. Haemost..

[168] Jayandharan G.R., Shaji R.V., Baidya S., Nair S.C., Chandy M., Srivastava A. (2005). Molecular characterization of factor IX gene mutations in 53 patients with haemophilia B in India. Thromb. Haemost..

[169] Thompson A.R., Schoof J.M., Weinmann A.F., Chen S.H. (1992). Factor IX mutations: Rapid, direct screening methods for 20 new families with hemophilia B. Thromb. Res..

[170] von Heijne G. (1985). Signal sequences. J. Mol. Biol..

[171] Bristol J.A., Freedman S.J., Furie B.C., Furie B. (1994). Profactor IX: The propeptide inhibits binding to membrane surfaces and activation by factor XIA. Biochemistry.

[172] Kaufman R. (1998). Post-translational Modifications Required for Coagulation Factor Secretion and Function. Thromb. Haemost..

[173] Liddell M.B., Lillicrap D.P., Peake I.R., Bloom A.L. (1989). Defective propeptide processing and abnormal activation underlie the molecular pathology of factor IX Troed-y-Rhiw. Br. J. Haematol..

[174] Montejo J.M., Magallón M., Tizzano E., Solera J. (1999). Identification of twenty-one new mutations in the factor IX gene by SSCP analysis. Hum. Mutat..

[175] Wulff K., Schröder W., Wehnert M., Herrmann F.H. (1995). Twenty-five novel mutations of the factor IX gene in haemophilia B. Hum. Mutat..

[176] Liu Z., Zhang Y., Zhu M., Zhang B. (2019). Identification of candidate nonsense mutations of FVIII for ribosomal readthrough therapy. Haematologica.

[177] Hartl F.U. (2002). Molecular Chaperones in the Cytosol: From Nascent Chain to Folded Protein. Science (80-).

[178] Sitia R., Braakman I. (2003). Quality control in the endoplasmic reticulum protein factory. Nature.

[179] Ciechanover A., Orian A., Schwartz A.L. (2000). Ubiquitin-mediated proteolysis: Biological regulation via destruction. BioEssays.

[180] Schröder M., Kaufman R.J. (2005). ER stress and the unfolded protein response. Mutat. Res. Mol. Mech. Mutagen..

[181] Muntau A.C., Leandro J., Staudigl M., Mayer F., Gersting S.W. (2014). Innovative strategies to treat protein misfolding in inborn errors of metabolism: Pharmacological chaperones and proteostasis regulators. J. Inherit. Metab. Dis..

[182] Engin F., Hotamisligil G.S. (2010). Restoring endoplasmic reticulum function by chemical chaperones: An emerging therapeutic approach for metabolic diseases. Diabetes Obes. Metab..

[183] Rajan R.S., Tsumoto K., Tokunaga M., Tokunaga H., Kita Y., Arakawa T. (2011). Chemical and Pharmacological Chaperones: Application for Recombinant Protein Production and Protein Folding Diseases. Curr. Med. Chem..

[184] Cortez L., Sim V. (2014). The therapeutic potential of chemical chaperones in protein folding diseases. Prion.

[185] Arakawa T., Timasheff S.N. (1985). The stabilization of proteins by osmolytes. Biophys. J..

[186] Brown C.R., Hong-Brown L.Q., Biwersi J., Verkman A.S., Welch W.J. (1996). Chemical chaperones correct the mutant phenotype of the ΔF508 cystic fibrosis transmembrane conductance regulator protein. Cell Stress Chaperones.

[187] Sato S., Ward C.L., Krouse M.E., Wine J.J., Kopito R.R. (1996). Glycerol Reverses the Misfolding Phenotype of the Most Common Cystic Fibrosis Mutation. J. Biol. Chem..

[188] Howard M., Welch W.J. (2002). Manipulating the Folding Pathway of ΔF508 CFTR Using Chemical Chaperones. Cyst. Fibros. Methods Protoc..

[189] Howard M., Fischer H., Roux J., Santos B.C., Gullans S.R., Yancey P.H., Welch W.J. (2003). Mammalian Osmolytes andS-Nitrosoglutathione Promote ΔF508 Cystic Fibrosis Transmembrane Conductance Regulator (CFTR) Protein Maturation and Function. J. Biol. Chem..

[190] Lim M., McKenzie K., Floyd A.D., Kwon E., Zeitlin P.L. (2004). Modulation of ΔF508 Cystic Fibrosis Transmembrane Regulator Trafficking and Function with 4-Phenylbutyrate and Flavonoids. Am. J. Respir. Cell Mol. Biol..

[191] Burrows J.A.J., Willis L.K., Perlmutter D.H. (2000). Chemical chaperones mediate increased secretion of mutant alpha 1-antitrypsin (alpha 1-AT) Z: A potential pharmacological strategy for prevention of liver injury and emphysema in alpha 1-AT deficiency. Proc. Natl. Acad. Sci. USA.

[192] Maegawa G.H.B., Tropak M.B., Buttner J.D., Rigat B.A., Fuller M., Pandit D., Tang L., Kornhaber G.J., Hamuro Y., Clarke J.T.R. (2009). Identification and Characterization of Ambroxol as an Enzyme Enhancement Agent for Gaucher Disease. J. Biol. Chem..

[193] Zimran A., Altarescu G., Elstein D. (2013). Pilot study using ambroxol as a pharmacological chaperone in type 1 Gaucher disease. Blood Cells, Mol. Dis..

[194] Porto C., Ferrara M.C., Meli M., Acampora E., Avolio V., Rosa M., Cobucci-Ponzano B., Colombo G., Moracci M., Andria G. (2012). Pharmacological Enhancement of α-Glucosidase by the Allosteric Chaperone N-acetylcysteine. Mol. Ther..

[195] Claudio M.G. (2013). Protein Misfolding in Disease and Small Molecule Therapies. Curr. Top. Med. Chem..

[196] Bernier V., Lagacé M., Bichet D.G., Bouvier M. (2004). Pharmacological chaperones: Potential treatment for conformational diseases. Trends Endocrinol. Metab..

[197] Loo T.W., Bartlett M.C., Clarke D.M. (2005). Rescue of Folding Defects in ABC Transporters Using Pharmacological Chaperones. J. Bioenerg. Biomembr..

[198] Johnson S.M., Connelly S., Fearns C., Powers E.T., Kelly J.W. (2012). The Transthyretin Amyloidoses: From Delineating the Molecular Mechanism of Aggregation Linked to Pathology to a Regulatory-Agency-Approved Drug. J. Mol. Biol..

[199] Pereira D.M., Valentão P., Andrade P.B. (2018). Tuning protein folding in lysosomal storage diseases: The chemistry behind pharmacological chaperones. Chem. Sci..

[200] Tao Y.-X., Conn P.M. (2018). Pharmacoperones as Novel Therapeutics for Diverse Protein Conformational Diseases. Physiol. Rev..

[201] Hou Z.-S., Ulloa-Aguirre A., Tao Y.-X. (2018). Pharmacoperone drugs: Targeting misfolded proteins causing lysosomal storage-, ion channels-, and G protein-coupled receptors-associated conformational disorders. Expert Rev. Clin. Pharmacol..

[202] Malhotra J.D., Miao H., Zhang K., Wolfson A., Pennathur S., Pipe S.W., Kaufman R.J. (2008). Antioxidants reduce endoplasmic reticulum stress and improve protein secretion. Proc. Natl. Acad. Sci. USA.

[203] Roelse J.C., De Laaf R.T.M., Timmermans S.M.H., Peters M., van Mourik J.A., Voorberg J. (2000). Intracellular accumulation of factor VIII induced by missense mutations Arg593Cys and Asn618Ser explains cross-reacting material-reduced haemophilia A. Br. J. Haematol..

[204] Roth S.D., Schüttrumpf J., Milanov P., Abriss D., Ungerer C., Quade-Lyssy P., Simpson J.C., Pepperkok R., Seifried E., Tonn T. (2012). Chemical Chaperones Improve Protein Secretion and Rescue Mutant Factor VIII in Mice with Hemophilia A. PLoS ONE.

[205] Guillet B., Lambert T., d’Oiron R., Proulle V., Plantier J.-L., Rafowicz A., Peynet J., Costa J.-M., Bendelac L., Laurian Y. (2006). Detection of 95 novel mutations in coagulation factor VIII geneF8responsible for hemophilia A: Results from a single institution. Hum. Mutat..

[206] Tjeldhorn L., Iversen N., Sandvig K., Bergan J., Sandset P., Skretting G. (2010). Functional characterization of the protein C A267T mutation: Evidence for impaired secretion due to defective intracellular transport. BMC Cell Biol..

[207] Chollet M.E., Skarpen E., Iversen N., Sandset P.M., Skretting G. (2015). The chemical chaperone sodium 4-phenylbutyrate improves the secretion of the protein CA267T mutant in CHO-K1 cells trough the GRASP55 pathway. Cell Biosci..

[208] Nagel-Wolfrum K., Möller F., Penner I., Baasov T., Wolfrum U. (2016). Targeting Nonsense Mutations in Diseases with Translational Read-Through-Inducing Drugs (TRIDs). BioDrugs.

[209] Yukihara M., Ito K., Tanoue O., Goto K., Matsushita T., Matsumoto Y., Masuda M., Kimura S., Ueoka R. (2011). Effective Drug Delivery System for Duchenne Muscular Dystrophy Using Hybrid Liposomes Including Gentamicin along with Reduced Toxicity. Biol. Pharm. Bull..

[210] Bidou L., Allamand V., Rousset J.-P., Namy O. (2012). Sense from nonsense: Therapies for premature stop codon diseases. Trends Mol. Med..

[211] Dabrowski M., Bukowy-Bieryllo Z., Zietkiewicz E. (2018). Advances in therapeutic use of a drug-stimulated translational readthrough of premature termination codons. Mol. Med..

[212] Batshaw M.L., Tuchman M., Summar M., Seminara J. (2014). A longitudinal study of urea cycle disorders. Mol. Genet. Metab..

